# The influence of molecular mobility on the properties of networks of gold nanoparticles and organic ligands

**DOI:** 10.3762/bjnano.5.177

**Published:** 2014-09-29

**Authors:** Edwin J Devid, Paulo N Martinho, M Venkata Kamalakar, Úna Prendergast, Christian Kübel, Tibebe Lemma, Jean-François Dayen, Tia E Keyes, Bernard Doudin, Mario Ruben, Sense Jan van der Molen

**Affiliations:** 1 Huygens-Kamerlingh Onnes Laboratory, Leiden Institute of Physics, Leiden University, Niels Bohrweg 2, 2333 CA Leiden, The Netherlands; 2 Institute of Nanotechnology, Karlsruhe Institute of Technology (KIT), Hermann-von-Helmholtz-Platz 1, 76344 Eggenstein-Leopoldshafen, Germany; 3 Centro de Química e Bioquímica (CQB), Faculdade de Ciências, Universidade de Lisboa, Campo Grande, 1749-016 Lisboa, Portugal; 4 Department of Microtechnology and Nanoscience, Chalmers University of Technology, SE-41296 Göteborg, Sweden; 5 School of Chemical Science, Dublin City University (DCU), Dublin 9, Ireland; 6 Karlsruhe Nano Micro Facility (KNMF) Hermann-von-Helmholtz-Platz 1, 76344 Eggenstein-Leopoldshafen, Germany; 7 Université de Strasbourg, IPCMS-CMRS UMR 7504, 23 Rue du Loess, 67034 Strasbourg, France

**Keywords:** aromatic capping ligands, gold nanoparticles, molecular charge transport, self-assembly, surface enhanced Raman spectroscopy

## Abstract

We prepare and investigate two-dimensional (2D) single-layer arrays and multilayered networks of gold nanoparticles derivatized with conjugated hetero-aromatic molecules, i.e., *S*-(4-{[2,6-bipyrazol-1-yl)pyrid-4-yl]ethynyl}phenyl)thiolate (herein S-BPP), as capping ligands. These structures are fabricated by a combination of self-assembly and microcontact printing techniques, and are characterized by electron microscopy, UV–visible spectroscopy and Raman spectroscopy. Selective binding of the S-BPP molecules to the gold nanoparticles through Au–S bonds is found, with no evidence for the formation of N–Au bonds between the pyridine or pyrazole groups of BPP and the gold surface. Subtle, but significant shifts with temperature of specific Raman S-BPP modes are also observed. We attribute these to dynamic changes in the orientation and/or increased mobility of the molecules on the gold nanoparticle facets. As for their conductance, the temperature-dependence for S-BPP networks differs significantly from standard alkanethiol-capped networks, especially above 220 K. Relating the latter two observations, we propose that dynamic changes in the molecular layers effectively lower the molecular tunnel barrier for BPP-based arrays at higher temperatures.

## Introduction

Inspired by nature, self-assembly is a bottom-up method to fabricate structures at all scales from nanometer-sized ingredients. In this way, new functional materials can be created with properties that are, in principle, based on the specific functionality of their building blocks [1]. An interesting approach, used for molecular conductance experiments, includes nanoparticles (ca. 10 nm) incorporated to bridge the size gap between macroscopic electrodes (larger than 100 nm) and molecules (ca. 1 nm) [2–8]. Typically, 2D arrays of gold nanoparticles capped by alkanethiols are created, after which dithiolated conjugated molecules are allowed to form molecular bridges between neighboring nanoparticles [6,8]. Although molecular insertion cannot be driven to completeness for thermodynamic reasons [9–10], this protocol has proven successful in molecular electronics, e.g., by providing access to switchable molecular devices [11–12]. The process can also be used for non-thiol ligands [13]. Here, we extend this self-assembly procedure beyond alkanes, making use of an attractive class of molecular ligands.

Molecules of the tridentate 2,6-bi(pyrazolyl)pyridine (BPP) group are well known to act as weak σ-donor/π-acceptor ligands exhibiting an octahedral coordination environment with a coordination number of six for transition metals [14]. Moreover, in the case of iron(II) ions, the BPP-ligands adjust the ligand field strength to access the so-called ST or spin crossover (SCO) regime [15], in which the physical properties depend strongly on their intrinsic low and high spin states (LS, S = 0 and HS; S = 2). Integrated spin transition (ST) units may be considered as potential electronic components in the construction of switching molecular devices [16–17], a vision for which the control of the attaching of BPP-units to gold nanoparticles sets the stage. Towards this goal, the synthetic introduction of substituents at the 4’-position of the pyridine moiety of BPP has been shown to be a useful strategy. In particular, the introduction of highly conductive π-conjugated phenylethynyl linker moieties with acetyl-protected thiol anchoring groups facilitates the contact to noble and coinage metal electrodes [18].

**Scheme 1:**
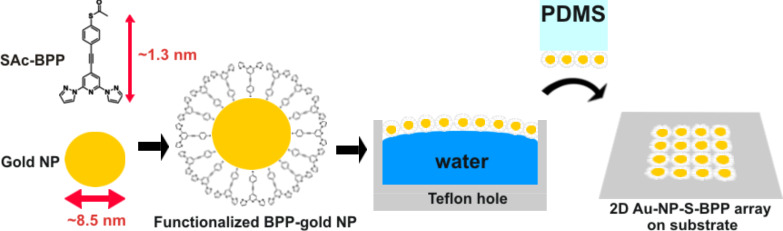
Fabrication of 2D Au-NP–S-BPP array (not to scale). The ingredients are gold nanoparticles (diameter 8.5 ± 1.5 nm) and SAc-BPP molecules (length of the molecule 1.3 nm, without the acetyl (Ac) group). The arrays are fabricated through self-assembly and then placed onto a substrate of choice by PDMS microcontact printing.

In this study, we report on the fabrication of 2D single-layer ligand–gold nanoparticle arrays (and multilayer ligand–gold nanoparticle networks) formed by gold nanoparticles covered by planar aromatic organic ligand-based molecules, namely *S*-(4-{[2,6-bipyrazol-1-yl)pyrid-4-yl]ethynyl}phenyl)thiolate (S-BPP). The inclusion of the thioacetate end group (see SAc-BBP molecule, Scheme 1) [18] is expected to steer the adsorption of the S-BPP molecule to the gold nanoparticles [19]. The results of the structural and spectroscopic characterisation of the synthesized 2D ligand-gold nanoparticle arrays (in short Au-NP–S-BPP-arrays), by means of UV-vis and electron microscopy (SEM, HRTEM and 3D TEM) experiments, will be presented. Specifically, surface enhanced Raman spectroscopy (SERS) provides insight into the selectivity of the bond formation. Remarkably, Raman experiments also reveal subtle shifts in some S-BPP modes related to reversible structural modification within the array induced by temperature. This observation is compared to temperature-dependent transport experiments. For this purpose, the fabricated 2D Au-NP–S-BPP arrays are electrically contacted to lithographically defined devices [5,8–9,12] and the obtained conductance measurements are compared to benchmark networks formed with alkanethiols spacers.

## Experimental

### Capping of gold nanoparticles with S-BPP molecules

To create arrays of S-BPP-capped gold nanoparticles, the well-established procedure to make alkanethiol-based nanoparticle arrays [8–9,20] is adapted and applied. The first step is the synthesis of citrate-stabilized gold nanoparticles in aqueous solvent (see Supporting Information File 1, experimental section (part 1)). This is followed by centrifuging 5 mL of monodispersed citrate-stabilized gold nanoparticle suspension at 15000 RPM for 1 h. After centrifugation, the aqueous solvent is removed from the settled gold nanoparticles, followed immediately by the redispersion of the nanoparticles in ethanol. Through forceful shaking, the nanoparticles are redispersed quickly to yield a stable colloidal dispersion, which is sealed in a glass bottle and sonicated in a water bath for approximately one hour.

Functionalization of gold nanoparticles with S-BPP molecules [18] is accomplished in two steps. First, 14.5 mg of S-BPP molecules is added to a sealable glass bottle with 2 mL of ethanol and heated in a water bath to 70 °C, under stirring until complete dissolution of the S-BPP molecules. Second, the hot S-BPP ethanolic solution is quickly added to the ethanolic dispersion of gold nanoparticles and sealed with a lid. The process of functionalization is accelerated by sonication in a water bath. After 15 min, the dispersion of functionalized gold nanoparticles has changed colour. As shown below, UV–visible absorption spectroscopy reveals that this colour change can be attributed to the S-BPP molecule assembling around the gold nanoparticles. Next, the functionalized gold nanoparticles are left to settle down by gravity in a cold, dark storage environment [21–22] for three days. The supernatant is then removed from the sediment, which is redispersed in 4 mL of chloroform and sonicated for 1 h. Finally, a lightly purple coloured dispersion of functionalized gold nanoparticles is used to prepare a self-assembled 2D Au-NP–S-BPP array.

### Fabrication of 2D Au-NP–S-BPP arrays

The 2D single-layer Au-NP–S-BPP array is prepared by using a Langmuir–Schaefer (LS) method (Scheme 1) in which a single-layer array of functionalized gold nanoparticles is self-assembled at the air–water interface [8,23–24] (see Supporting Information File 1, Figure S5). A Teflon mould, containing a hole, is filled with 300 μL of demineralised Millipore water. Then, 30 μL of S-BPP functionalized gold nanoparticles in chloroform are dispersed on the aqueous layer. The steadily evaporating chloroform leaves the nanoparticles at the air–water interface. The hydrophobically functionalized gold nanoparticles attract each other on the water surface and self-assemble into a highly ordered array. The array is then transferred onto a Si wafer substrate (covered with 300 nm of SiO_2_) by using a polydimethylsiloxane (PDMS) microcontact printing method [8]. Other suitable types of substrates for the microcontact printing of these samples are glass, quartz, several types of plastics (like polyethylene, polypropylene foils and polyimide (Kapton) films) and electron beam lithography-written high-aspect-ratio (HAR) nanotrench electrodes devices [25]. The Au-NP–S-BPP arrays are stored in a dark and cold environment and can be kept for several months.

## Results and Discussion

### Imaging of Au-NP–S-BPP arrays and networks

Scanning transmission electron microscopy (SEM) is used to image the arrays on flat (oxidized) silicon substrates and the Au-NP–S-BPP networks on nanotrench electrodes devices (see Supporting Information File 1, Figure S8). Scanning transmission electron microscopy (STEM) and transmission electron microscopy (HAADF-STEM, HRTEM and 3D TEM) are also used to accurately characterise the nanoscale structuring of the multilayered networks on carbon-covered TEM grids. It should be noted that, whereas regular 2D structures are readily obtained on flat (oxidized) silicon substrates, the ordered assembly on the TEM grids turned out to be more challenging, resulting in structures with local ordering only.

**Figure 1:**
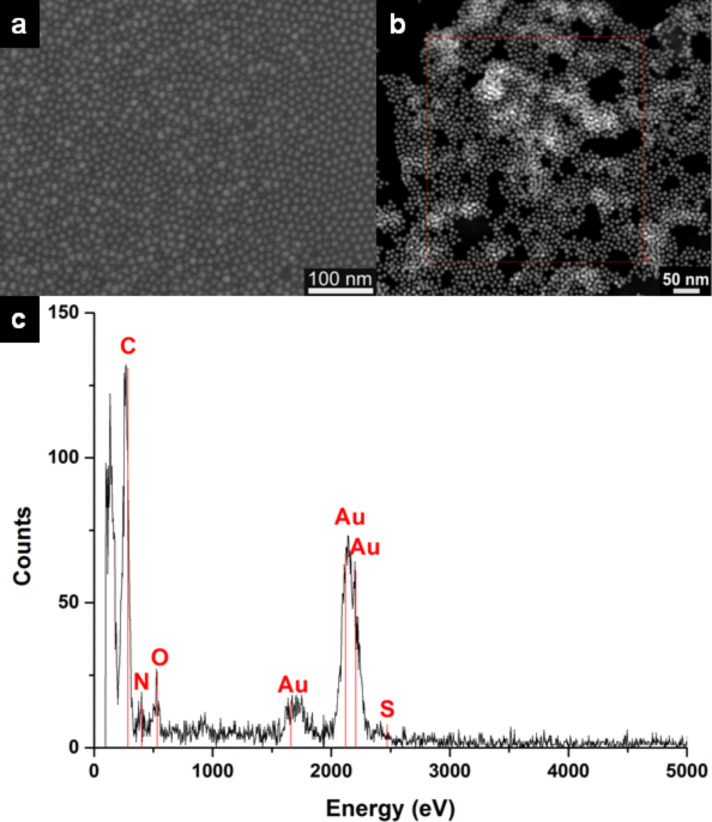
Characterization of Au-NP–S-BPP arrays and networks by electron microscopy. a) SEM image of a 2D single-layer microcontact printed Au-NP–S-BPP array on a flat Si–SiO_2_ substrate; b) STEM-reference image of a Au-NP–S-BPP network area on a TEM grid substrate; c) local EDX analysis revealing the elemental composition.

Figure 1a shows a SEM image of a nanoparticle array that was microcontact printed on a SiO_2_ substrate. Typically, these Au-NP–S-BPP arrays reveal ordered structures on flat surfaces extending over several hundred nanometers, depending on the microcontact printing procedure used. The nanoparticles have not coalesced as they are well-separated by the capping ligands S-BPP.

HAADF-STEM images of Au-NP–S-BPP networks (see Figure 1b and Supporting Information File 1, Figure S6) show a fairly uniform distribution of functionalized gold nanoparticles, sometimes as a single layer, but typically as a few stacked layers. The latter is probably caused by repeating deposition of Au-NP–S-BPP arrays on the TEM grids by dipping the TEM grids. Microscopic analyses of the network confirm the monodispersity of the gold nanoparticles with an average diameter of 8.5 ± 1.5 nm. Moreover, fast Fourier transforms (FFT) of the HAADF-STEM images indicate an average particle distance of 10.6 nm. The resulting average next neighbour distance is close to 2 nm, consistent with an expected value between one (1.3 nm) and two (2.6 nm) molecular lengths of S-BPP.

Figure 1c shows the energy dispersive X-ray (EDX) spectrum corresponding to the HAADF-STEM image of the thin Au-NP–S-BPP network film (see Figure 1b). In addition to Au, the expected spectra of the organic ligand elements C, N and S (partially overlapped by Au) are detected. The observed oxygen peak may originate from the presence of water when dipping the TEM grid into the self-assembled Au-NP–S-BPP arrays that floats on water. HAADF-STEM tomography [26–27] is used to further analyse the spatial packing of multilayered Au-NP–S-BPP networks (see Supporting Information File 1, Figure S7). From a 3D reconstruction of a multilayered Au-NP–S-BPP network, we find indeed that the S-BPP functionalized gold nanoparticles not only form a packed array separated by organic S-BPP molecules in 2D, but also enable sterically driven ordering of gold nanoparticles layers in a partial multi-stack volume.

### UV–vis spectroscopy

Ultraviolet-visible (UV–vis) spectroscopy is performed to gain insight into the optical properties of these molecule–gold nanoparticle arrays, and specifically to investigate the influence of the S-BPP molecules on the effective dielectric constant. Metal nanoparticles exhibit absorbance due to surface plasmon resonances (SPR) that occur at frequencies ω (or wavelengths λ) at which the surface charges of the particles are oscillating resonantly with the electromagnetic driving field (the incoming photons). For nearly spherical particles, only one SPR mode is expected [28]. According to the Mie theory in the dipolar quasi-static approximation (in which the diameter *d* of the nanoparticle is assumed to be much smaller than the wavelength, i.e., *d* << λ), the position of the SPR is directly related to the permittivity of the medium surrounding the nanoparticle [29–30]. In molecule–gold nanoparticle arrays, this permittivity will be largely influenced by the molecules separating the nanoparticles. Hence, Au-NP–S-BPP arrays are expected to have optical properties different from reference alkanethiol–gold nanoparticle arrays. A further influence on the SPR is exerted by the optical interaction of one nanoparticle with the full set of neighbouring gold nanoparticles.

A suitable way to describe the SPR in molecule–gold nanoparticle arrays is through the Maxwell–Garnett theory (again in the quasi-static approximation). This effective medium theory defines an effective dielectric constant of the medium ε_eff_ that takes into account both the presence of the surrounding medium and the neighbouring nanoparticles [31–33]. The resonance condition is then given by:

[1]
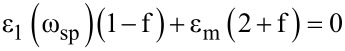


Here, ω_sp_ denotes the frequency of the SPR and ε_m_ is the dielectric constant of the medium surrounding the nanoparticles. The so-called filling factor f = *V*_clusters_/*V*_total_ denotes the relative volume occupied by other nanoparticles around the resonating nanoparticle. In this way, the surrounding nanoparticles in an array are incorporated into the theory effectively. Note that for f = 0 (i.e., there is no interaction between the nanoparticles) the condition is ε_1_(ω) = −2ε_m_, as in standard Mie theory.

**Figure 2:**
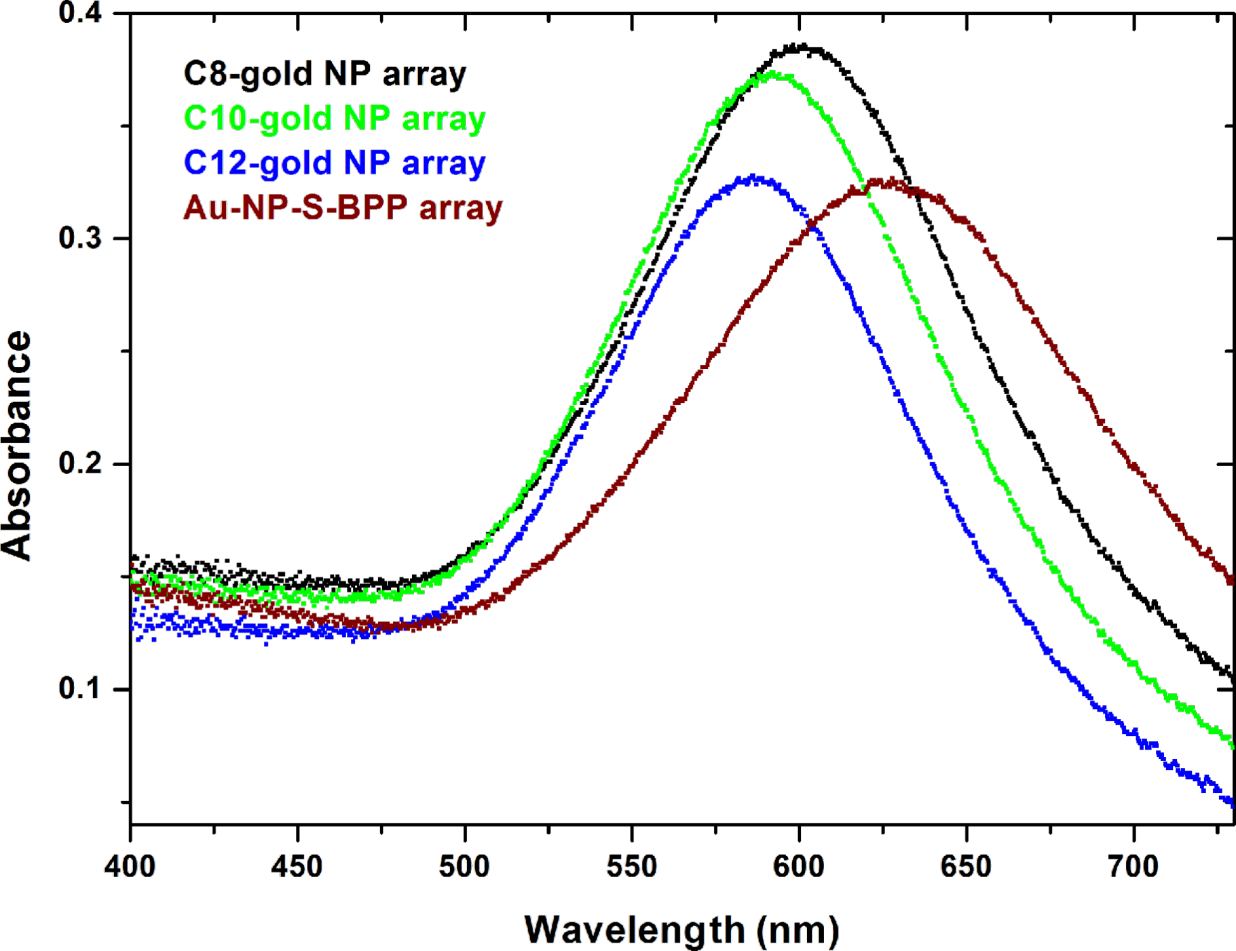
Surface plasmon resonance spectroscopy of several functionalized gold nanoparticle arrays studied in our work. Gold nanoparticles are covered by C8 (black), C10 (green), C12 (blue) and S-BBP (red), respectively.

Figure 2 shows the absorption curves for four types of 2D molecule–gold nanoparticle arrays, with alkanethiol-protected gold nanoparticles of various lengths (C8, C10, C12) compared to the 2D Au-NP–S-BPP array. From C8 to C12, the SPR shifts to shorter wavelengths [9], as expected for a blue shift originating from a change in the spacing distance between the gold nanoparticles, i.e., a decrease in the filling factor f, (see Equation 1). Note that the change in ε_m_ is expected to be negligible for the alkanethiol series [30,34]. However, the situation is different for the 2D Au-NP–S-BPP array, where we do expect a significant change in ε_m_, since S-BPP molecules are fully conjugated and hence more easily polarizable. Compared to the alkanethiol–gold nanoparticle arrays, a red shift is indeed observed in Figure 2. This is also confirmed experimentally from UV–vis spectroscopy of a C8–gold nanoparticle dispersion and a S-BPP–gold nanoparticle dispersion (both in chloroform), in which effectively f = 0. In Figure S9 (see Supporting Information File 1) we show that the SPR peak of the S-BPP-covered gold nanoparticles in solution is red-shifted by 17 nm compared to the C8-gold nanoparticle dispersion. By using Mie theory (f = 0), we estimate the relative dielectric constants for C8–gold nanoparticle dispersion and S-BPP–gold dispersion to be 2.2 ± 0.1 and 2.8 ± 0.1, respectively.

The results obtained so far can now be checked for consistency. Since we are able to estimate f from the electron microscopy images, for both the C8–gold nanoparticle array and the Au-NP–S-BPP array, we can apply the Maxwell–Garnett theory to estimate the dielectric constant ε_m_ in these arrays as well. Indeed, we find approximately the same values for ε_m_ within an array or in dispersion, for both S-BPP molecules and for C8 molecules, summarized in Table 1. These values are consistent with the values for alkanethiol–gold nanoparticle arrays and oligo phenylene ethynylene (OPE)-bridged gold nanoparticle arrays obtained through molecular exchange [9,30]. We note that the latter type of arrays does contain a mixture of OPE molecules and alkanes, unlike our Au-NP–S-BPP arrays.

**Table 1:** Relative dielectric constant due to the ligands ε_m_ (octanethiols vs BPP ligands) as calculated from UV–vis spectra of both nanoparticle solutions and nanoparticle arrays.

type of functionalized gold nanoparticles	f	relative dielectric constant ε_m_

C8–gold nanoparticle dispersion	0	2.2 ± 0.1
S-BPP–gold nanoparticle dispersion	0	2.8 ± 0.1
C8–gold nanoparticle array	0.35	2.4 ± 0.1
Au-NP–S-BPP array	0.36	2.8 ± 0.1

### Room-temperature Raman spectroscopy of 2D Au-NP–S-BPP arrays

**Figure 3:**
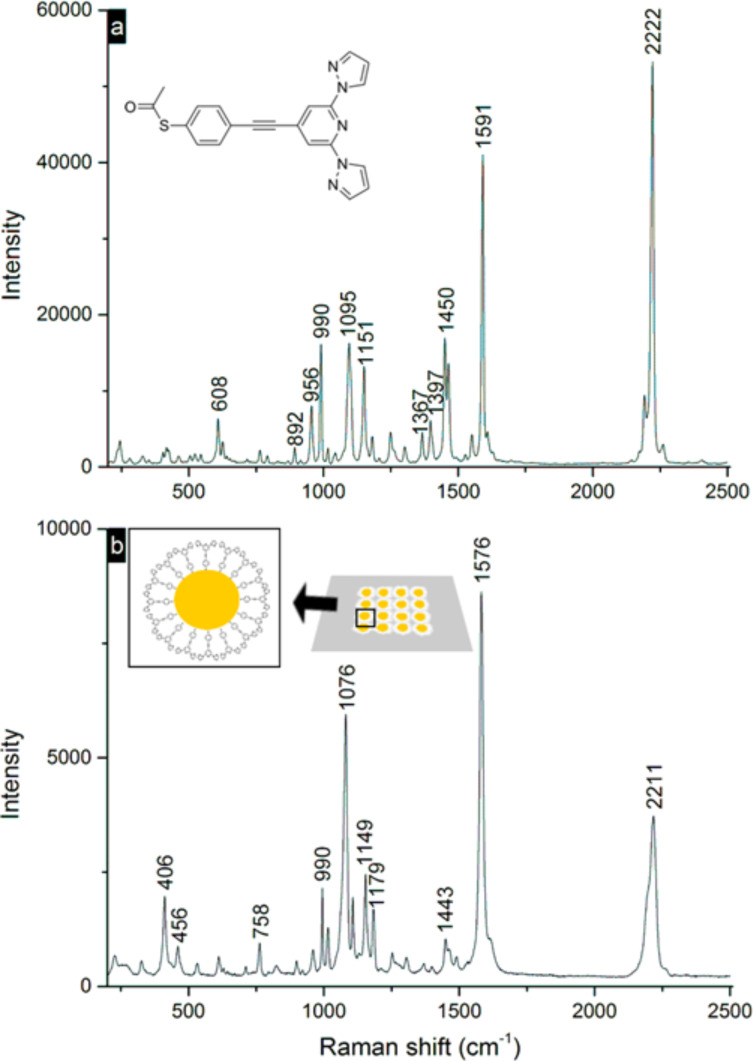
a) Room temperature Raman spectrum of bulk (powder) SAc-BPP molecules showing the region of 200–2400 cm^−1^ excited at 633 nm (1.2 mW illuminating power on the sample); b) Room temperature Raman spectrum of 2D (single layer) Au-NP–S-BPP array obtained by microcontact printing on a quartz substrate.

In order to get more insight into the binding of the S-BPP molecules to the gold as well as to investigate the temperature-dependence of the molecular ordering, surface enhanced Raman spectroscopy of the 2D Au-NP–S-BPP array was performed. In Figure 3 the room temperature Raman spectra of a bulk (powder) sample of S-BPP molecules (see Figure 3a) and of a 2D Au-NP–S-BPP array (see Figure 3b) are compared. In spite of the much lower concentration of the S-BPP molecule anticipated from the 2D single layer coated array (at least three orders of magnitude) compared to the bulk value, the Raman spectral intensity is comparable between the two types of samples (see Figure 3a vs Figure 3b). Moreover, the Raman spectra from the Au-NP–S-BPP array show a better signal-to-noise ratio attributed to surface enhancement of the Raman signal. The arrayed nature of the gold nanoparticles and their small interparticle separation leads to a plasmon absorbance shown in Figure 2, which is resonant with the 633 nm excitation used here. Although there are clear commonalities, the spectra of the 2D single layer array and SAc-BPP powder spectra exhibit notable differences. The individual spectral features are broader in the SERS spectrum, consistent with the higher heterogeneity in the microenvironment the S-BPP molecules experienced in the array compared to the powder. Furthermore, the SERS spectrum is less complex than that of the powder. This is consistent with plasmonic enhancement as the vibrational modes involving the atoms closest to the gold nanoparticle will be selectively enhanced.

Whereas the low frequency Au–S or Au–N stretch modes cannot be easily discerned from the background in the spectral region below 400 cm^−1^, it is interesting to note that the thioacetate and acetate modes observed in the solid sample, for example a weak feature at 1698 cm^−1^ assigned to the acetate C=O and features between 1367–1380 cm^−1^, are no longer evident in the SERS spectrum of the Au-NP–S-BPP arrays. This strongly suggests that the thiol is bound to the gold after surface-mediated hydrolysis of the acetate group. The dominance of key benzenethiol modes in the SERS spectrum is also indicative of binding via thiol moiety. The most intense Raman feature in the powder spectrum of S-BPP is the aryl in-plane C–C stretch mode centred at 1591 cm^−1^. This mode is shifted to 1576 cm^−1^ in the bound S-BPP molecule of the 2D arrays, which matches precisely the in-plane C–C stretch reported for SERS of benzenethiol on copper or silver and is a further indication of binding through sulfur or thioacetate [35–36].

The second most intense feature in the SERS spectrum is a mode at 1076 cm^−1^, which is assigned to the aromatic C–S stretch shifted from 1095 cm^−1^ in the powder sample. Both the shift and enhancement of this mode is characteristic of SAMs of benzenethiol on plasmonic metals, and is further evidence that the S-BPP ligand is binding to the gold surface through this moiety [35–36]. Other characteristic benzenethiol features are also enhanced, at 990, 660 and 406 cm^−1^. The alkyne C≡C stretch mode, which is by far the most intense mode in the powder sample, is reduced in relative intensity in the 2D single-layer array, but remains a dominant feature albeit shifted from 2222 to 2211 cm^−1^ on surface binding. This suggests a modest weakening of the C≡C bond presumably induced by binding of the thiol to the surface [37]. By comparison, the Raman modes from the S-BPP moiety are weaker than the benzenethiol moiety in the SAM spectrum. The features at 1443, 1149, 1179 and 785 cm^−1^ are attributed to the S-BPP ligand, principally to the pyrazole moieties [38]. A weak shoulder centred at 1607 cm^−1^ is attributed to the pyridine moiety. The low relative intensity of this mode suggests it is not directly bound to the nanoparticle. Overall, the predominance of SERS signal from the benzenethiol moiety and the comparatively weaker enhancement of pyrazole modes provide strong evidence that the S-BPP ligand binds to the nanoparticles preferably through the thiol linker.

### Temperature-dependent Raman spectroscopy on 2D Au-NP–S-BPP arrays

**Figure 4:**
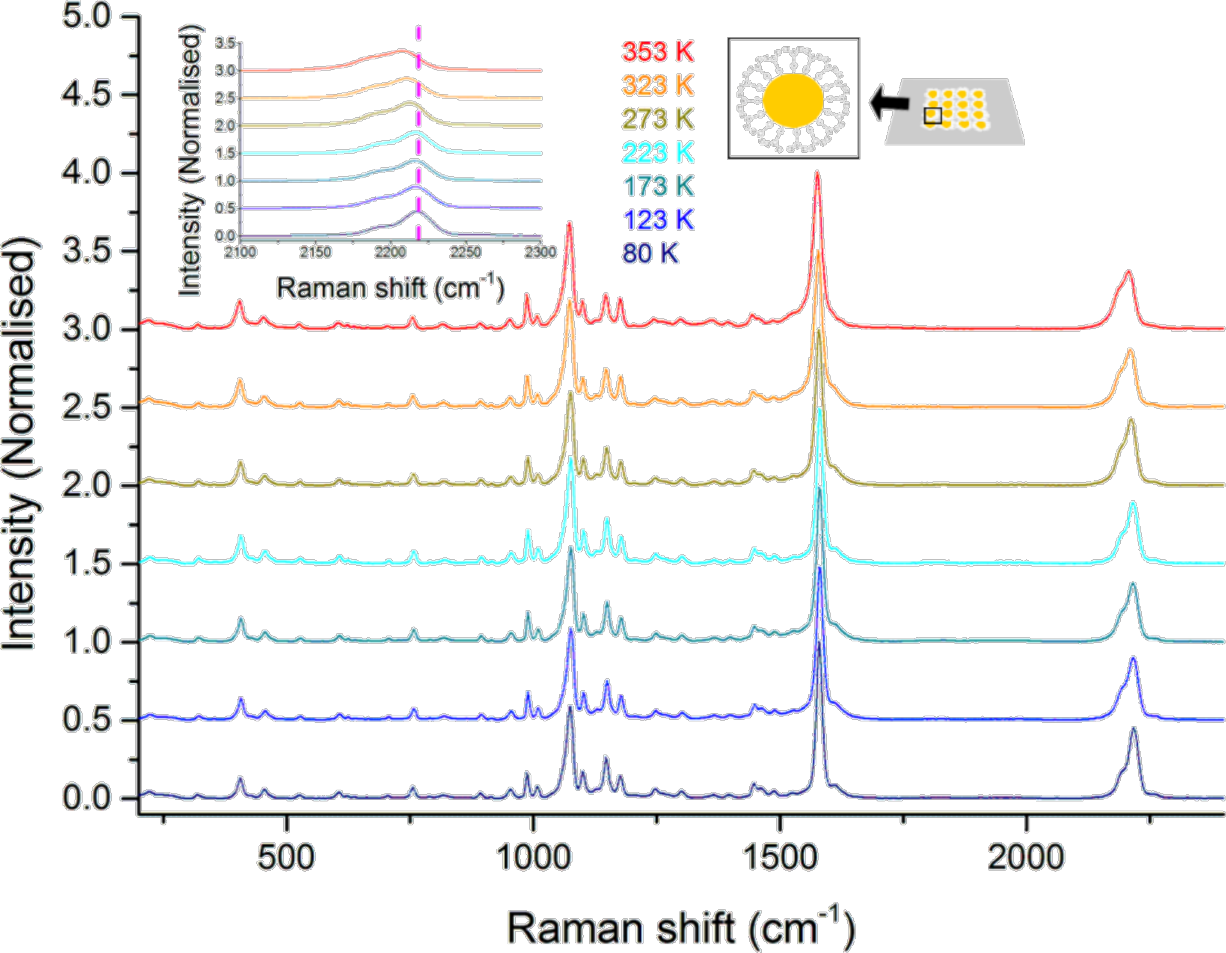
Temperature-dependent Raman spectra of 2D Au-NP–S-BPP arrays microcontact printed on a quartz substrate excited at 633 nm (illuminating power 1.2 mW at the sample). Inset: expansion of the alkyne stretch mode; the vertical marker emphasizes the temperature shifts in the spectra.

The studies by Raman microscopy over the temperature range 80 to 353 K are shown in Figure 4. The absence of significant chemical changes with temperature is indicated by the overall similarity between the spectra under temperature variation. However, some subtle changes can be noticed. Across most of the SERS-enhanced modes, a small shift to the blue of between 2 and 4 cm^−1^ is observed with decreasing temperature. Interestingly, the unaffected modes are the weakest features in the spectrum, i.e., those that are not strongly SERS enhanced. In other words the bonds nearest to the nanoparticle surface are most affected by the changing temperature. This observation suggests that temperature induces changes to bonding interactions between the nanoparticle and BPP. Possible changes are conformational/orientational changes of BPP with respect to the nanoparticle surface, which might be expected to cause shifts in the frequencies of bonds close to the nanoparticle. Such electronic changes would also be likely to transmit to chemical moieties conjugated to the bound group [39]. Correspondingly, the most strongly affected modes are the C–C stretch associated with the benzenethiol at 1576 cm^−1^ at room temperature, which shifts to 1582 cm^−1^ at 80 K and to 1575 cm^−1^ at 353 K. The alkyne C≡C stretch mode is also particularly strongly affected and shifts from a value of 2207 cm^−1^ at 353 K to 2211 cm^−1^ at room temperature, and to 2223 cm^−1^ at 80 K. This mode is broad at 353 K, comprising a main feature and a shoulder at room temperature, which sharpens and resolves at 80 K into a second band at 2190 cm^−1^. As described previously, a significant shift in the C≡C stretch mode was observed on thiol binding to the gold surface indicating the significant electronic coupling between the benzenethiol and the surface. It is noteworthy that the C≡C stretch mode is so sensitive to temperature in this system, as also found in other reports on Raman of alkynes [37]. Their large polarizability renders them sensitive to electronic changes within their molecular vicinity, particularly in conjugated systems [40–41]. The surface-bound benzenethiol is sensitive to temperature, but the alkyne exhibits much larger temperature-dependent spectral changes, with a shift of 16 cm^−1^ between 80 and 353 K. We therefore tentatively attribute this to changes in the orientation of the molecule, possibly promoted by Au–S mobility at the surface with temperature. It is important to note that the Raman spectral changes with temperature are fully reversible, i.e., restoring the Au-NP–S-BPP arrays to room temperature after a cooling to 80 K leads to a recovery of the original room spectrum with all features.

Comparison studies on alkanethiol-modified gold nanoparticle arrays reveal that temperature changes in their associated Raman spectra are much more modest than those observed for Au-NP–S-BPP arrays (see Supporting Information File 1, Figure S10). The largest shifts observed in individual modes typically did not exceed 2 cm^−1^ and sharpening (improved resolution) of the vibrational bands, was the main effect of low temperature. This observation appears to validate our hypothesis that molecular structural/orientational changes related to the S-BPP ligand bound to the nanoparticle are driving the changes observed in the Raman spectrum with temperature. We note that both the magnitude and the reversibility of the Raman changes observed with temperature effectively preclude the possibility that the actual binding mode to the nanoparticle is changing. The extensive lateral interactions characteristics of alkanethiol–nanoparticle arrays make them more tightly packed than S-BPP. Therefore S-BPP is more prone to random orientation and has more freedom to reorient at the nanoparticle surface promoted by temperature. The observed spatial flexibility of the thiol-anchored BPP ligands is of interest for molecule chelation purposes, as it should facilitate the envisioned complexation of Fe(II) metal ions.

### Conductance measurements on a multilayered Au-NP–S-BPP network

Charge transport in Au-NP–S-BPP network devices provides additional experimental insight into the question of thermal stability of the nanoparticles architecture. Here, not only the S-BPP molecules, but also the nanoparticles are expected to play a role. At low temperatures, the thermal energy *k*_B_*T* becomes comparable to their charging energy *E*_C_ = *e*^2^/2*C*, i.e., the electrostatic energy needed to add an electron onto the metallic nanoparticle (here *C* is the total capacitance of a nanoparticle in the array and *e* is the electron charge). In that case, Coulomb blockade will hamper charge transport. Earlier work on alkanethiol–gold nanoparticle networks demonstrated a wide range of charge transport behaviours within the Coulomb-blockade regime [2,5,9,42]. We have recently studied the cross-over between the sequential tunneling and cotunneling regimes for alkanethiol networks, as well as for alkanethiol networks with dithiolated OPE-3 bridges [5]. We estimated typical Coulomb-blockade charging energies of around 14–17 meV [5], in correspondence with temperature- and voltage-dependent transport measurements. Hence, in alkanethiol and OPE-based networks Coulomb blockade dominates below 200–250 K, whereas around room temperature, the current–voltage (*I*–*V*) characteristics are linear and practically independent of temperature.

The same method as in [5] is used to investigate the charge transport through Au-NP–S-BPP networks. We fabricate nanotrench devices with a high width-to-length aspect ratio (ca. 200) by electron beam lithography and metal lift-off. Through patterning Ti(3 nm)/Au(47 nm)-electrodes of 20 μm width, separated by a gap of around 100 nm (about 10 nanoparticles), are created on Si/SiO_2_ substrates. We transfer the nanoparticle array onto these electrodes via a PDMS stamp. Samples containing 2D single-layer Au-NP–S-BPP arrays typically exhibited very high resistance values (more than 100 GΩ). For that reason, we decided to stamp multilayers of Au-NP–S-BPP networks (three times stamped). Note that the data shown below are obtained after full stabilization of the sample, following an initial resistance decrease as a function of time at room temperature. Most likely, the latter is the result of a slow re-ordering process [43–45].

**Figure 5:**
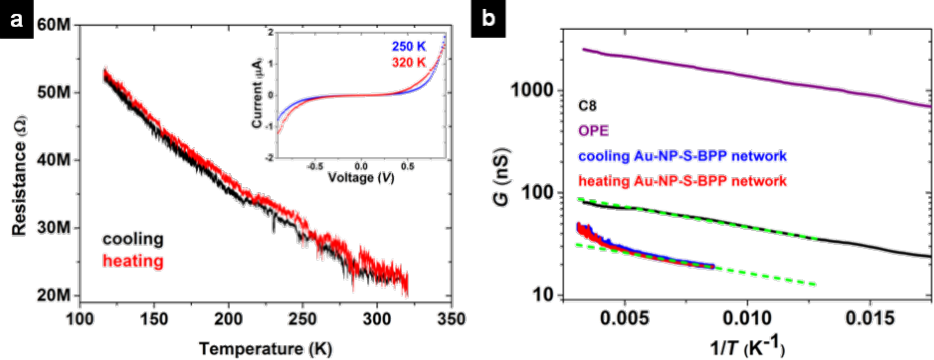
a) Low-bias resistance of a multilayered Au-NP–S-BPP network as a function of the temperature for 116 K < *T* < 320 K. Both cooling (black) and heating (red) traces are shown. Inset shows *I*–*V* curves of multilayered Au-NP–S-BPP network at 250 K and 320 K; b) Arrhenius plot (semilog plot of *G* vs 1/*T*) of the data in Figure 5a compared to experiments on similar, i.e., three times stamped, C8 networks (black) and OPE-bridged networks (purple). The green dashed lines are parallel guides to the eye to compare the slope of various networks.

Figure 5a shows the low-bias resistance vs temperature for a multilayered Au-NP–S-BPP network microcontact printed on a nanotrench device. The interparticle voltage bias, typically smaller than the device voltage bias by a factor of 10, is of the order of 3 mV. We find that the resistance decreases monotonically with temperature over the entire temperature range, without a clear saturation at higher temperatures (see the inset of Figure 5a and Supporting Information File 1, Figure S11 for temperature dependent current–voltage (*I*–*V*) curves of Au-NP–S-BPP networks).

If we compare octanethiol and OPE-based networks with the Au-NP–S-BPP network, the latter behaves differently at higher temperatures. For example, for the first two types of samples, the low-bias resistance is basically independent of voltage and temperature at room temperature, indicating that *k*_B_*T*_R_ > *E*_C_ (see Supporting Information File 1, Figure S12) [5]. However, the *I*–*V* curves of the Au-NP–S-BPP network are surprisingly non-linear, with large currents occurring at larger bias. We focus our discussion on the low-bias behaviour, in the linear approximation. The particular temperature-dependent behaviour of the Au-NP–S-BPP networks becomes most clear in Figure 5b, which shows the data of Figure 5a in an Arrhenius plot. For comparison, we also display Arrhenius plots for C8 and OPE networks (taken from Supporting Information File 1, Figure S12), rather well-described by activated behaviour between 70 K and 300 K as indicated by the straight-line approximation in Figure 5. This is indeed expected for the regime of sequential tunnelling (note that for the lower-temperature cotunneling regime, Efros–Shklovskii behaviour is expected, resulting in an exponential dependence on *T*^−0.5^ [2,42]). For the three types of samples, there is now a clear similarity in the low-temperatures range, illustrated by the parallel lines in the semilog plot of Figure 5b, indicating an activation energy of the same order for the three molecular spacers. Nevertheless, the Au-NP–S-BPP network sample exhibits a very different behaviour for higher temperatures, showing a clear upturn in the Arrhenius plot (i.e., at lower 1/*T* values in Figure 5b).

A first explanation for a deviation might be that the charging energy *E*_C_ = *e*^2^/2*C* 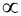 *C*^−1^ is actually higher for the Au-NP–S-BPP networks than for the alkanethiols networks. This would be the case if the nanoparticle radius *R* and/or the effective dielectric constant ε_eff_ around the nanoparticles were considerably lower for the Au-NP–S-BPP networks than for the octanethiol networks (cf. the self-capacitance of a sphere: *C* = 4πε_0_ε_r_*R*, see also Supporting Information File 1, Figure S13 for models including nearest neighbours). However, this is not likely. First, the nanoparticles are made in the same manner for all sets of devices (independent of the molecular species investigated) and they are thus observed to be of similar size. Second, UV–vis spectroscopy indicates that ε_m_ (and also ε_eff_ which incorporates f) is actually higher for Au-NP–S-BPP networks than for alkanethiol networks (see Table 1). This should thus lead to a lower charging energy for S-BPP than for octanethiol networks and hence to linear *I*–*V*-curves at room temperature. Additionally, it is difficult to reconcile a Coulomb-blockade picture with the continuous increase of the slope of the Arrhenius plot of Figure 5b when heating Au-NP–S-BPP networks. The latter would suggest a significant change of the activation energy, i.e., of the charging energy, as temperature increases. For these reasons, we exclude Coulomb blockade as the reason for the upturn in Figure 5b.

Following the results from temperature-dependent Raman spectroscopy, we propose that the deviation from simple Arrhenius-law behaviour originates from fluctuations in the molecular interconnects, resulting in a modification of the effective tunnel barrier. Indeed, the nanoparticles are separated by loosely interacting S-BPP molecules, which cannot form a close-packed structure and hence keep room for thermally-driven motions. At higher temperatures, variations in the relative orientation of S-BPP neighbours can result in fluctuations in π–π interactions, yielding changes in charge transfer probability between nanoparticles. We expect that in this situation, the effective transmission of the tunnel barrier becomes the time-average of the set of all possible configurations, each with their own specific transmission value, in a way somewhat similar to the recent proposal in [46]. For example, temporarily enhanced π–π interaction should lead to higher tunnelling probabilities. Time-averaging of such fluctuations may thus result in an enhanced transport at high temperatures, explaining the upturn in the Arrhenius plot for the S-BPP network in Figure 5b. One should point out that the transport measurements per se do not present a conclusive evidence of disorder-enhanced conductivity of nanoparticles networks. However, the combination of Raman spectroscopy and transport measurements favours such a model, proposing a new approach for understanding how disorder can impact the transport properties in molecular junctions. Additional calculations will be required to extend our hypothesis of fluctuations-enhanced transport between particles, taking into account the percolation character of transport in molecularly interconnected arrays. Complementary future experiments may use conducting-probe AFM or eutectic GaIn methods. In that case, monolayers of S-BPP formed at both electrodes could be gently brought into mechanical contact, after which temperature-dependent I(V) measurements can be done.

## Conclusion

Self-assembled gold nanoparticle arrays, stabilized by a new type of conjugated organic molecules as capping ligands, can be assembled into two-dimensional arrays that form locally well-ordered structures on different types of substrates. Raman spectroscopy reveals that these S-BPP molecules coordinate selectively to the nanoparticles through thiol–gold bonds, leaving the pyridine and pyrazole available for a further chemical binding. Temperature-dependent Raman measurements exhibit frequency shifts for several key modes of the S-BPP molecules, which points to changes of the molecular orientation occurring at high temperatures. The extent of structural dynamics is far greater than observed for simple alkanethiol-modified nanoparticle arrays, and is consistent with the less dense packing anticipated for the S-BPP ligands. This provides also an explanation for the peculiar temperature dependence of the electrical properties of the S-BPP networks, which shows a clear deviation from Arrhenius behaviour above 220 K. Hence, our work suggests that the conductance behaviour of molecule–nanoparticle arrays can be tuned as an indicator of dynamical disorder in these structures, which can be a prerequisite to create nanoparticle-network candidates for further chemical functionalization or reactivity. This sets the stage for the attractive possibility of a coordination with Fe(II) metal ions, thereby introducing switchable spin transition units into the network.

## Supporting Information

File 1 Additional experimental data.
