# Supplementary material for: The influence of molecular mobility on the properties of networks of gold nanoparticles and organic ligands
Source: Beilstein J Nanotechnol. 2014 Sep 29;5:1664–74. doi: 10.3762/bjnano.5.177 (PMC4222375; doi:10.3762/bjnano.5.177)
Supplement: File 1 — Additional experimental data. [file Beilstein_J_Nanotechnol-05-1664-s001.pdf]

Supporting Information

for

The influence of molecular mobility on the properties of networks of gold nanoparticles and organic ligands

Edwin J. Devid[§1], Paulo N. Martinho[§2,3], M. Venkata Kamalakar[4], Úna Prendergast[5], Christian Kübel[2,6], Tibebe Lemma[5], Jean-François Dayen[7], Tia. E. Keyes*[5], Bernard Doudin*[7], Mario Ruben*[2,7] and Sense Jan van der Molen*[1]

Address: [1]Huygens-Kamerlingh Onnes Laboratory, Leiden Institute of Physics, Leiden University, Niels Bohrweg 2, 2333 CA Leiden, The Netherlands; [2]Institute of Nanotechnology, Karlsruhe Institute of Technology (KIT), Hermann-von-Helmholtz-Platz 1, 76344 Eggenstein-Leopoldshafen, Germany; [3]Centro de Química e Bioquímica (CQB), Faculdade de Ciências, Universidade de Lisboa, Campo Grande, 1749-016 Lisboa, Portugal; [4]Department of Microtechnology and Nanoscience, Chalmers University of Technology, SE-41296 Göteborg, Sweden; [5]School of Chemical Science, Dublin City University (DCU), Dublin 9, Ireland; [6]Karlsruhe Nano Micro Facility (KNMF) Hermann-von-Helmholtz-Platz 1, 76344 Eggenstein-Leopoldshafen, Germany and [7]Université de Strasbourg, IPCMS-CMRS UMR 7504, 23 Rue du Loess, 67034 Strasbourg, France

E-mail: Sense Jan van der Molen* - Molen@Physics.LeidenUniv.nl; Mario Ruben - Mario.Ruben@kit.edu; Tia. E. Keyes - Tia.Keyes@dcu.ie; Bernard Doudin - Bernard.Doudin@ipcms.unistra.fr

* Corresponding author

[§] These authors contributed equally to this work

Additional experimental data

Outline:

1. Experimental section
2. Imaging of 2D Au-NP-S-BPP arrays and multilayered networks
3. Spectroscopic characterisation of alkanethiol-gold nanoparticles and S-BPP-gold nanoparticle dispersions
4. Surface enhanced Raman spectroscopic characterisation of alkanethiol-gold nanoparticles array
5. Charge transport data:

   - temperature dependent I-V’s of a multilayered Au-NP-S-BPP network

   - temperature dependant resistance of a C8-gold nanoparticle network and an OPE-gold nanoparticle network
6. Modelling the charging energy of 2D Au-NP-S-BPP array as function of the dielectric constant

1. Experimental Section

Part 1: Synthesis of charge stabilized monodispersed gold nanoparticles in water

Synthesis of monodispersed gold nanoparticles was made according to the following method. All solid reagents were purchased from Fluka and all organic liquid reagents were mainly purchased from Sigma Aldrich. The reagents were used in the state as they were received and some were diluted to the desired concentration. Reagents used and purchased from other suppliers will be mentioned. Further during the whole synthesis process (preparation of gold nanoparticles and 2D Au-NP-S-BPP arrays) we used Millipore demineralized water.

Monodispersed spherical gold particles with a diameter ~10 nm in water were synthesized through use of a method described by Slot and Geuze [1]. Charge stabilized gold nanoparticles (~10^{12} particles/mL) were prepared through reduction of gold(III) chloride hydrate ($HAuCl_4 \cdot 4H_2O$, purchased at Sigma Aldrich, 99.999% pure) in Millipore water. First 1 mL of $HAuCl_4$ • $4H_2O$ (1 % (w/v)) solution was added in a Erlenmeyer containing 79 mL Millipore water under stirring through use of magnetic stirrers (250 RPM). Second the reducing agents (all 1 % (w/v) solutions) namely 4 mL sodium citrate tribasic dehydrate (> 99.5%) and 80 µl tannic acid were solved in 16 mL Millipore water also under stirring (250 RPM). Both the solution of dissolved gold ions and the solution of reducing agents were heated to 60 °C under continuous stirring through a water bath on a hot plate. The solutions were temporarily sealed with glass stops or parafilm (purchased at Pechiney Plastic Packaging Company) to limit the evaporation of water from both solutions. When the solutions were at 60 °C, the solution of heated reducing agents was rapidly added to the heated gold ions

solution under vigorously stirring. The mixture of gold ions with reducing agents was kept at 60 °C for 2 minutes.

Then the Erlenmeyer containing the reduced gold ions was connected to the condenser to return the evaporating water from the mixture during boiling. The water bath was removed and the Erlenmeyer with reduced gold was heated on a hot plate further toward 100 °C. When the reduced gold ion mixtures show first signs of boiling, the mixture was continually heated for 10 minutes at boiling temperature. The dark purple coloured reduced gold ions mixture became a ruby red coloured dispersion of gold nanoparticles. Then the stirrer was activated during boiling and set at 250 RPM. After heating the gold nanoparticle dispersion the mixture was cooled down to room temperature under stirring (250 RPM) while still connected to the condenser setup. Through use of a cold water bath the cool down process can be speeded up. At the end a ruby red coloured dispersion of gold nanoparticles was stored in a bottle in the fridge (~7 °C).

Part 2: Synthesis of S-(4-{[2,6-Bipyrazol-1-yl)pyrid-4-yl]ethynyl}phenyl) ethanethioate (SAc-BPP)

The SAc-BPP molecule was obtained by a cross-coupling reaction of 4-ethynyl-2,6-di(pyrazol-1-yl)pyridine with 4-iodo-1-(thioacetyl)benzene using Sonogashira conditions. The procedure to synthesize the SAc-BPP molecule has been published elsewhere [2]. To obtain a higher yield the procedure was adapted and is described below.

4-Ethynyl-2,6-di(pyrazol-1-yl)pyridine (0.9936 g, 4.2 mmol), 4-iodo-1-(thioacetyl)benzene (1.2985 g, 4.6 mmol), $Pd(PPh_3)_2Cl_2$ (118.3 mg, 0.17 mmol, 4%)

and CuI (66.8 mg, 0.35 mmol, 8%) were placed into a 100 mL Schlenk tube, and the mixture was purged with argon several times. To this mixture were added freshly distilled THF (40 mL) and diisopropylamine (10 mL), and the reaction mixture was stirred at room temperature for 3 days. A brown solution and a dark orange precipitate were obtained. The precipitate was eliminated by filtration and 2 mL of anhydric acetic was added to the resulting brown solution. Solvents were removed with vacuum by rotary evaporation. The obtained brown oil product was purified by column chromatography on silica gel using a mixture of ethyl acetate/hexane (1:9) as eluent. The third colourless fraction showing a blue fluorescent spot on the TLC contains the desired product. After evaporation of solvents, the yellowish product was washed with diethyl ether, giving a white powder (685.7 mg, yield 42%).

^{1}H NMR (see Supporting Information, Figure S1) **(500 MHz, $CDCl_3$, 25 ºC, δ/ppm):** 8.54 (d, 2H, pyrazole), 7.96 (s, 2H, pyridine), 7.77 (s, 2H, pyrazole), 7.58 (d, 2H, phenyl), 7.44 (d, 2H, phenyl), 6.50 (s, 2H, pyrazole), 2.45 (s, 3H, $-CH_3$). **^{13}C NMR (**see Supporting Information, Figure S2) (**125 MHz, $CDCl_3$, 25 ºC, δ/ppm**): 193.14, 150.35, 142.72, 136.38, 134.40, 132.69, 129.75, 127.22, 123.10, 111.50, 108.30, 94.12, 88.04, 30.46. **ESI-TOF MS** (see Supporting Information, Figure S3) in $CHCl_3$ + formic acid (Da): *m/z* (rel. intensity, assigned structure) = 386.15 (100%, $C_{21}H_{16}N_5OS$, **M**+H^+, calc. = 386.11), 408.14 (22%, $C_{21}H_{15}N_5NaOS$, **M**+Na^+). **FT-IR** (see Supporting Information, Figure S4) **(KBr):** ν/cm^{-1} = 3108, 2917, 2219, 2190, 1695, 1628, 1607, 1552, 1525, 1469, 1449, 1397, 1349, 1318, 1298, 1260, 1208, 1123, 1103, 1095, 1042, 1034, 1017, 991, 955, 917, 894, 863, 837, 826, 792, 763, 750, 716, 631, 624, 610, 598, 546, 504, 463, 424.

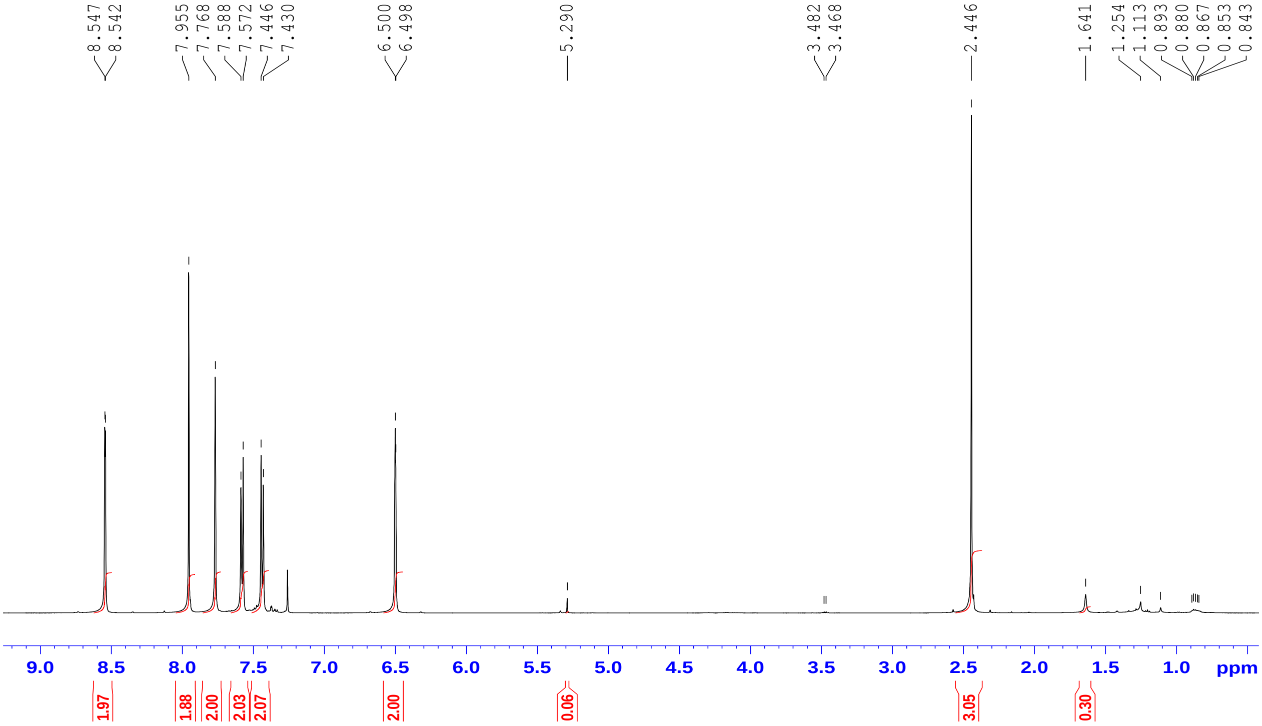

Figure S1: ^{1}H NMR spectrum of S-(4-{2,6-(dipyrazol-1-yl)pyrid-4-yl)]ethynyl}phenyl) ethanethioate (SAcBPP) in $CDCl_3$.

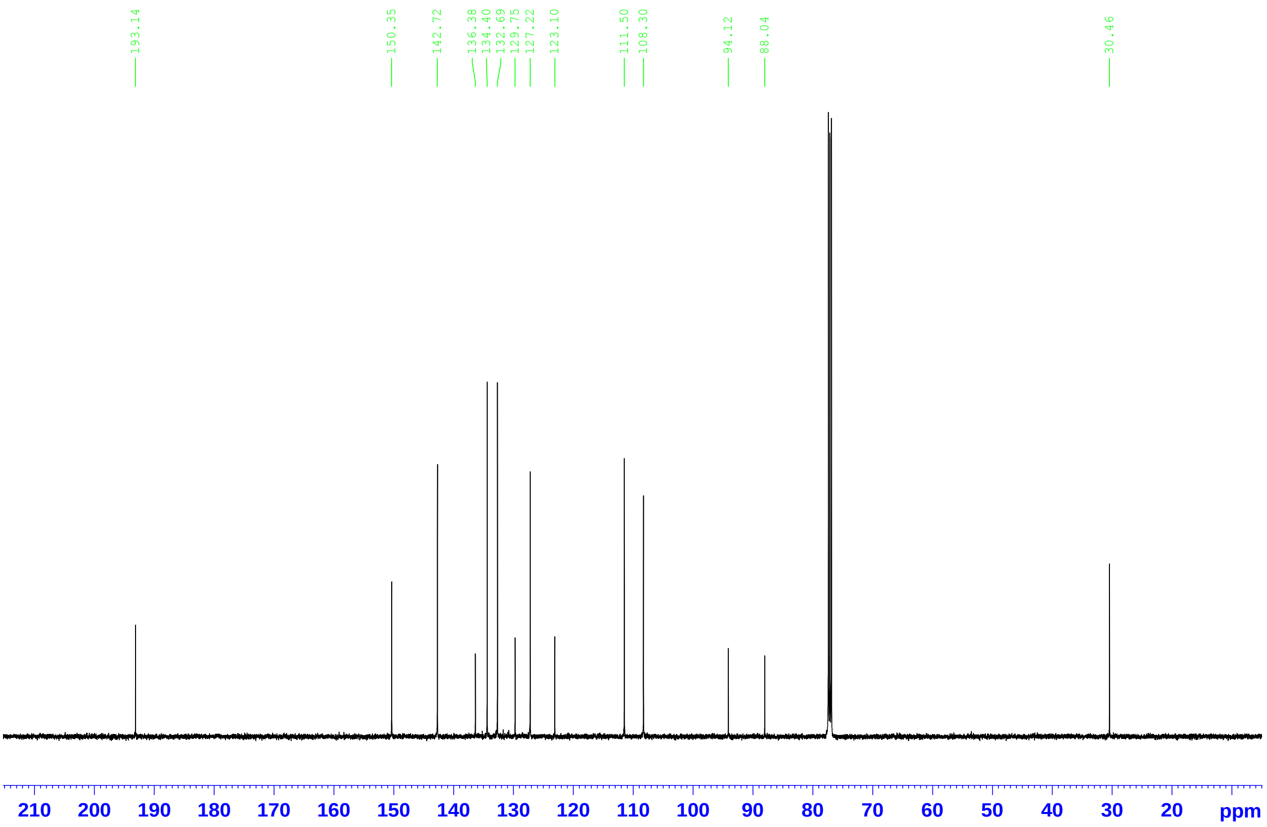

Figure S2: ^{13}C NMR spectrum of S-(4-{2,6-(dipyrazol-1-yl)pyrid-4-yl)]ethynyl}phenyl) ethanethioate (SAcBPP) in $CDCl_3$.

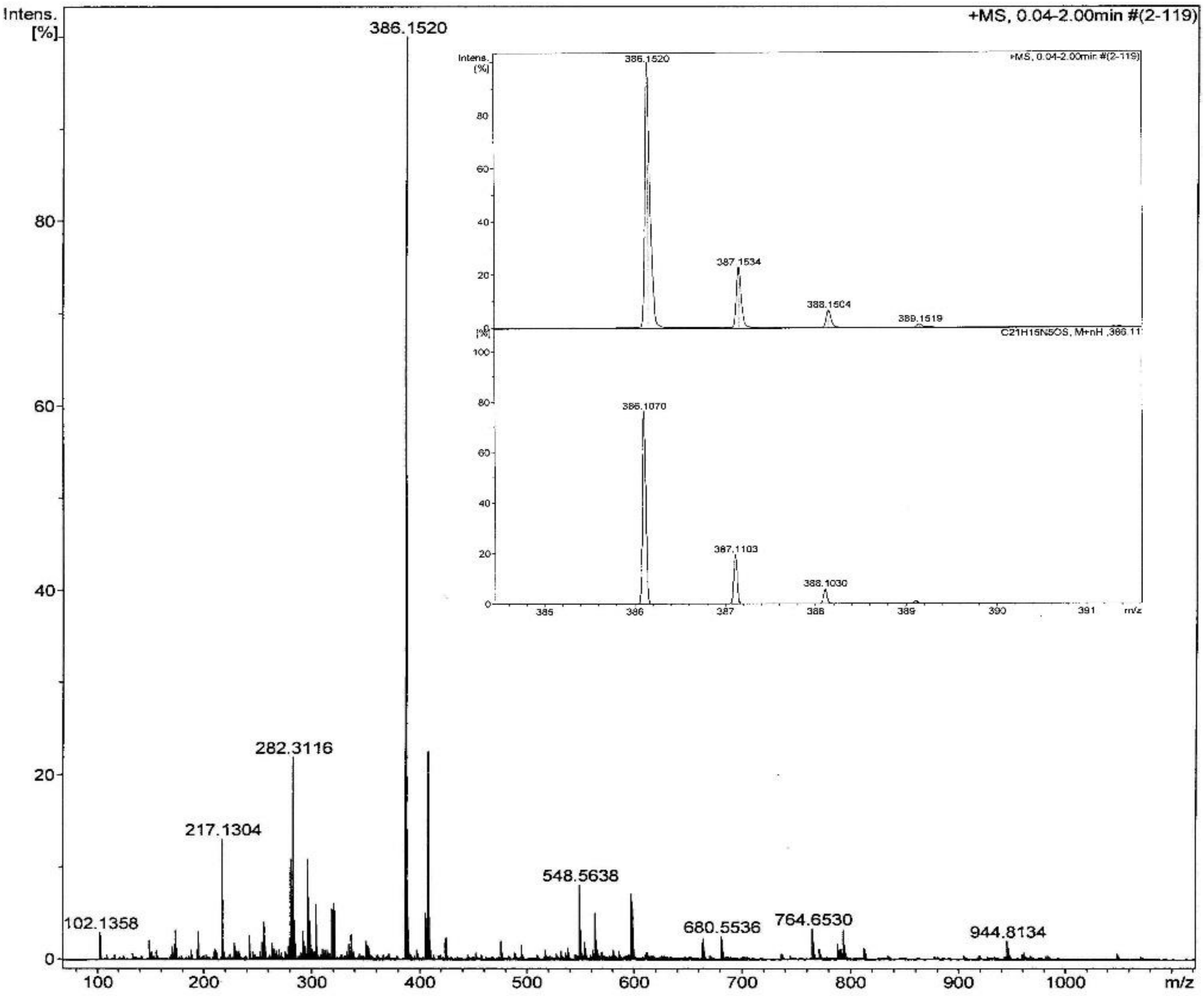

Figure S3: ESI-TOF mass spectrum of S-(4-{2,6-(dipyrazol-1-yl)pyrid-4-yl)]ethynyl}phenyl). (SAc-BPP)

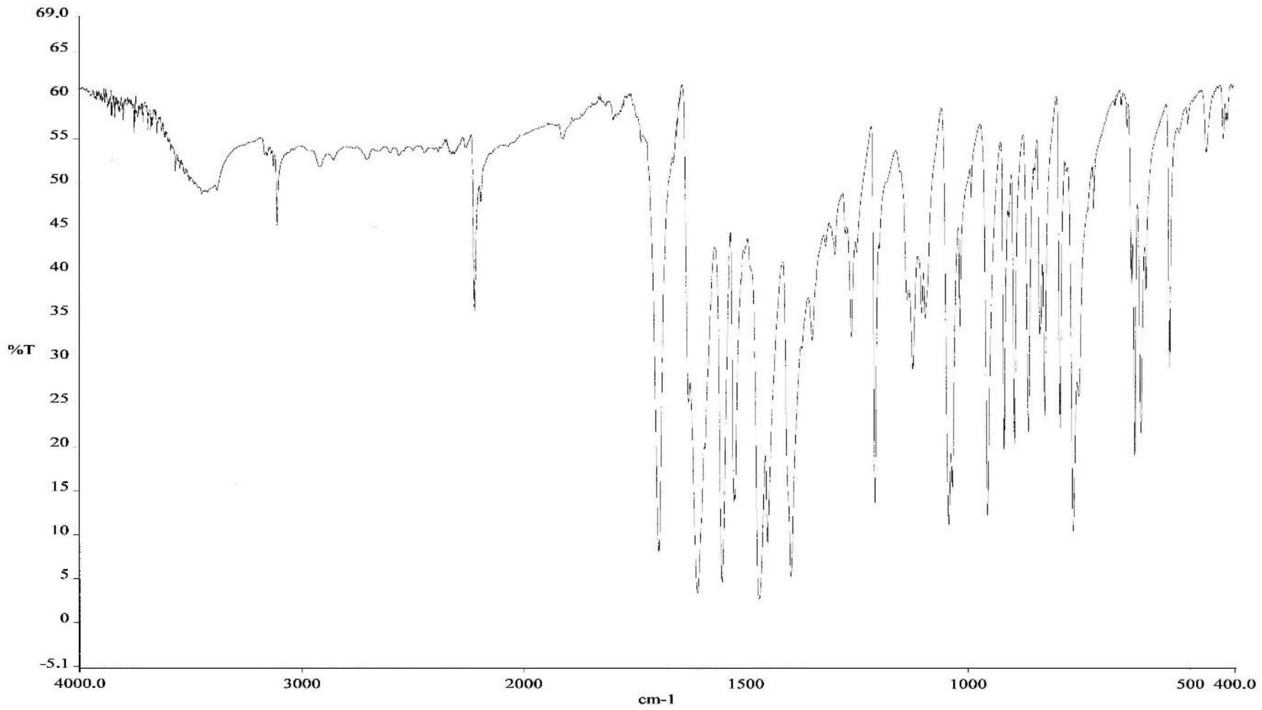

Figure S4: FT-IR spectrum of S-(4-{2,6-(dipyrazol-1-yl)pyrid-4-yl)]ethynyl}phenyl). (SAcBPP)

Part 3: Preparation of 2D Au-NP-S-BPP array

The functionalization of gold nanoparticles through SAc-BPP molecules and solvent exchange was performed through an developed spin-off method based on the method from Huang et al. [3]. Five Eppendorf tubes containing each 1 mL of charge stabilized gold nanoparticles in water were centrifuged at 15000 RPM for 1 hour at 10 ºC.

In the meantime a heated SAc-BPP solution was prepared for addition later to the gold nanoparticles in ethanol (in the described method was used ethanol absolute, purchased at Sigma Aldrich). 14.5 mg of SAc-BPP molecules were added into a glass bottle containing 2 mL of ethanol. This SAc-BPP solution was heated on a hot plate to 70 ºC, where the SAc-BPP solution was continuously stirred by stirring magnets (~250 RPM) to aid the dissolution of the SAc-BPP molecules. Within an hour the SAc-BPP molecules will be dissolved in hot ethanol and this was kept constant until the solvent exchange process was ready for the gold nanoparticles.

After centrifugation the gold nanoparticles were settled down and the supernatant (water) was removed through use of micropipettes. Then quickly 1 mL ethanol (absolute, purchased at Sigma Aldrich) was pipetted in the Eppendorf tube to redisperse the settled down gold nanoparticles in ethanol by shaking. The redispersed gold nanoparticles in ethanol were checked on their colour (showing a light pink/purple colour after redispersion) and if no aggregates were sticking to the Eppendorf tube (additionally redisperse them by putting the Eppendorf tube in an ultrasonic bath (Branson 1510).

All the five Eppendorf tubes with redispersed gold nanoparticles in ethanol were collected in a glass bottle (type DURAN, purchased at Sigma Aldrich) and sealed by

a screwable lid. The glass bottle with gold nanoparticles in ethanol was treated with the ultrasonic bath for at least 5 minutes to redisperse further the gold nanoparticle dispersion. After the sonic bath treatment the dispersion of gold nanoparticles in ethanol was quickly heated up through a hot plate to ~50 ºC. When the dispersion of gold nanoparticles in ethanol was warmed up, this bottle together with the bottle of heated up SAc-BPP solution were both removed from the hot plate. With care but quick the heated SAc-BPP solution was added to the warm gold nanoparticle dispersion. The mixture of gold nanoparticles with SAc-BPP molecules in ethanol was then placed for 5 minutes at ~50 ºC under continuously stirring (250 RPM). Then the mixture of gold nanoparticles with SAc-BPP molecules in ethanol was left to cool down to 20 ºC at rest. Preferably the dispersion of gold nanoparticles functionalized with SAc-BPP molecules was moved and saved in a fridge (~7 ºC) to promote further the settling down of the functionalized gold nanoparticles. After at least 3 days the gold nanoparticles functionalized with SAc-BPP molecules have settled down in an ethanol solvent.

Then the ethanol supernatant was removed by micropipette from the settled down functionalized gold nanoparticles with SAc-BPP molecules. The sediment of functionalized gold nanoparticles was redispersed in 2 mL chloroform (99 % pure, purchased at Boom chemicals) and the sealed bottle was placed in an ultrasonic bath for 1 hour. After sonication, a lightly purple coloured dispersion of functionalized gold nanoparticles with SAc-BPP molecules was obtained in chloroform and this dispersion was used to prepare self-assembled 2D Au-NP-S-BPP arrays based on a method described by Santhanam and Andres [4].

In the hole of a Teflon block was pipetted 300 µl Millipore water. Then 30 μL of the gold nanoparticles functionalized with SAc-BPP molecules in chloroform were deposited on to the water layer. Immediately the thin 2D single layer of Au-NP-S-BPP

array was formed on top of the water after evaporation of the chloroform in a fume hood (see Supporting Information, Figure S5).

The self-assembled 2D Au-NP-S-BPP array was picked up using a PDMS stamp (holding the stamp with a tweezer) from the water layer in the Teflon hole. The PDMS stamp with the 2D Au-NP-S-BPP array layer was then blown dry with nitrogen gas. The 2D Au-NP-S-BPP array on the PDMS stamp was printed gently on a substrate for 10 seconds and then was the stamp removed from the substrate. An imprint of 2D Au-NP-S-BPP array was now attached on the substrate. The substrates with 2D Au-NP-S-BPP arrays were stored in a closed storage box and saved in an environment of ~20 ºC. Through a microcontact printing method a self-assembled 2D Au-NP-S-BPP array can be picked up and printed on a substrate (like flat (oxidized) silicon- , glass- , quartz- tiles or certain plastics). On a high aspect ratio (HAR) nanotrench electrodes device multiple layers (maximally 3 times of microcontact printing) of 2D Au-NP-S-BPP arrays were used. Through this multilayer is obtained a sufficient coupling of the Au-NP-S-BPP network between the gold electrodes, separated by a trench ~100 nm long (see Supporting Information, Figure S8).

Here was used polydimethylsyloxane (PDMS) stamps for microcontact printing that were fabricated through a Sylgard 184 silicone elastomer (purchased from Dow Corning). Through mixing a prepolymer gel with a curing agent (Sylgard 184) the PMDS mixture was formed and poured inside a master model of a rectangular shape. Through degassing the PDMS mixture at 20 ºC for at least 30 minutes, the bubbles from previous mixing will escape out of the PDMS mixture. The mixture was then baked in an oven at 60 °C for 90 minutes. The obtained silicone PDMS was cooled down and later removed from the master model. The silicone PDMS was cut into small cubic PDMS stamps of desired sizes. The prepared PDMS stamps were placed in a glass beaker containing ethanol (absolute). This beaker of PDMS stamps was

washed in ethanol through sonication by an ultrasonic bath for at least 15 minutes. The prepared PDMS stamps were dried with nitrogen gas and stored in a storage box to be ready for microcontact printing of 2D Au-NP-S-BPP arrays.

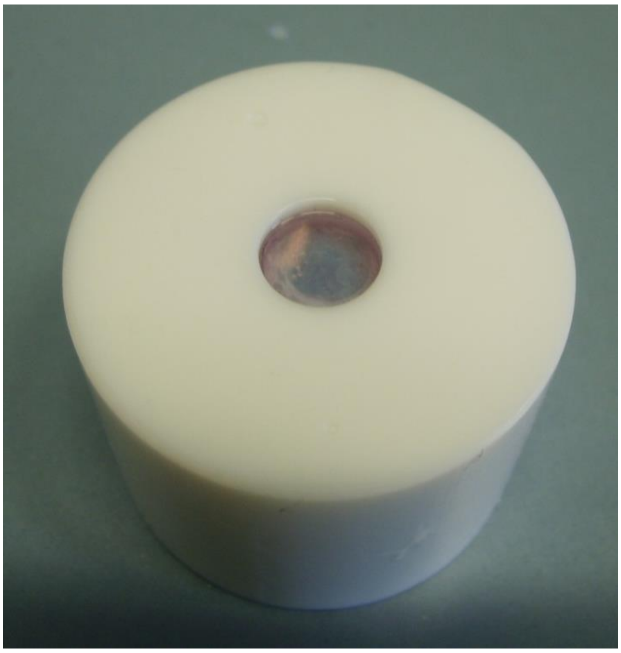

Figure S5: Picture of the self-assembly of a 2D Au-NP-S-BPP array at the water-air interface.

<u>Part 4: Used chemicals, used analysis methods (SEM, (3D)TEM), Raman, UV-Vis)</u>

TEM characterization of the Au-NP-S-BPP networks was performed using an image corrected FEI Titan 80-300 operated at 300kV, equipped with a Gatan US1000 slowscan CCD camera, an HAADF-STEM detector and an EDAX S-UTW EDX detector providing a nominal resolution of 0.8 Å for HRTEM and 1.4 Å for HRSTEM imaging. EDX analysis was performed averaging over an extended area with a nominal spot size of ~0.5 nm. Electron tomographic analysis was performed in HAADF-STEM mode with a spot size of ~0.3 nm and a pixel size of 0.36 nm over a

tilt-range of ±76º in 1.5º steps. The tilt-series was aligned using IMOD eTomo V4.5.1 with a residual mean error of 0.9 pixels. The 3D reconstruction was performed in Inspect3D V3.1 using the SIRT algorithm with 25 iterations. Visualization was done using IMOD and Amira V5.3.

The SEM images were obtained with the FEI NanoSEM200. The NanoSEM is a Schottky field emitter SEM that can obtain very high resolution down to low accelerating voltages (The resolution at 15 kV is 1 nm). Through a UHR immersion mode, where the focusing field(gradient) extends below the pole piece of the final lens, a specimen can be placed 'in' the lens at very small working distance. The UV-Vis measurements on 2D Au-NP-S-BPP arrays (and also alkanethiol-gold nanoparticle arrays, octanethiol functionalized gold nanoparticle dispersions and S-BPP-gold nanoparticle dispersions) were performed through an Ocean Optics Miniature Fiber Optic Spectrometer (model QE65000 spectrometer with UV-Vis-NIR light source Micropack DH-2000).

Micro-Raman spectroscopy was collected in backscattering configuration using an Olympus confocal microscope attached to a HORIBA Jobin-Yvon Labram HR 1000 spectrometer using a Peltier cooled (−70 ^{o}C) charge-coupled device (CCD) camera (255×1024 pixels), excited with vertically polarized 632.8 nm of a He-Ne laser. The spectrometer was equipped with diffraction gratings (600 and 1200 grooves/mm), and the slit set for 1 cm^{-1} spectral resolution. An appropriate edge filter was set in the spectrometer depending on the excitation source. The laser was focused with air objectives Olympus, 100x NA 0.9 or Leica, PL Fluotar L50x, NA 0.55. The laser spot on the samples was 1 μm in diameter and incident laser power at the sample was set between 1.2 mW. A LINKAN THMS 600 (Linkam Scientific Instruments Ltd) heating-cooling stage was used for temperature dependent measurements. And the sample was allowed to equilibrate for 20 minutes at each temperature before collection of a

spectrum. All spectra were collected n = 3 times. The Raman band of a silicon wafer at 520 cm^{-1} was used to calibrate the spectrometer along with the Rayleigh line. The spectral data were acquired and analyzed using LabSpec software (Labspec, Horiba/Jobin-Yvon Group).

Part 5: Preparation of devices and setups for conduction experiments

A layer of self-assembled gold nanoparticles, formed on water, is microcontact printed several times on a HAR nanotrench electrodes device [5] via microcontact printing [4] (see Supporting Information, Figure S8). The HAR nanotrench device (gap length ≈100 nm, gap width ≈20 μm) is fabricated using a combination of optical - and E-beam lithography. We used photolithography to make larger contact pads (by first deposition of gold contact pads on a Si-SiO_2 substrate), followed by E-beam lithography to write and link T or bow-shaped nanotrench electrodes to the already gold deposited contacts on the substrate. After e-beam assisted gold deposition and metal lift-off a gold HAR nanotrench (containing electrical interconnects electrodes) device is ready for stamping [5]. Low temperature electrical measurements were performed on the nanotrench devices in a liquid helium bath cryostat. The DC electrical properties of these networks are measured by Agilent semiconductor parametric E5270B analyzer.

2. Imaging of 2D Au-NP-S-BPP arrays and multilayered networks

Figure S6b shows a high resolution (HR)-TEM image of the gold nanoparticles in the Au-NP-S-BPP network. (on a TEM grid substrate). Most of the gold nanoparticles are not single crystalline, but consist a few crystalline domains, which are typically separated by twin boundaries. In addition to the well-known decahedral shaped gold particles, particles with various numbers of parallel twin boundaries are observed as well as some more complex defect structures, which cannot be clearly identified. The surface of the nanoparticles typically exhibits small, but well-defined facets.

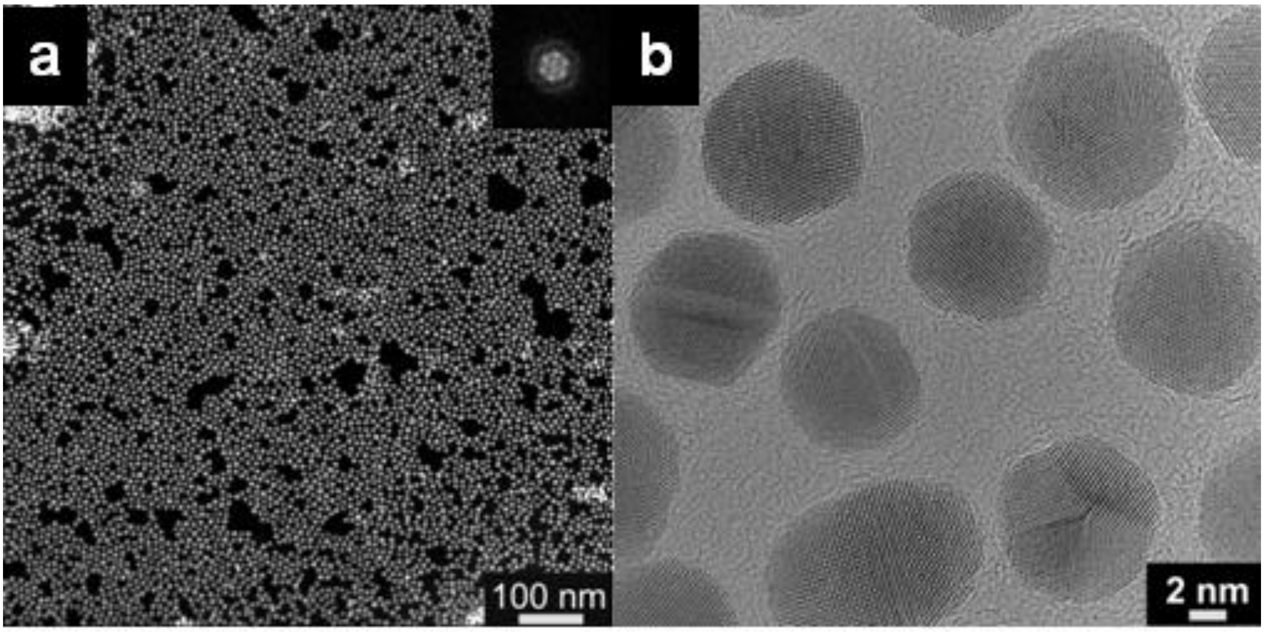

Figure S6: a) HAADF-STEM overview image revealing locally a hexagonal order (inset FFT) in an Au-NP-S-BPP network on a TEM grid substrate. From the FFT of 25 areas exhibiting a clear hexagonal order, the average particle distance determined as 10.6 nm. b) High resolution (HR)-TEM image of the gold nanoparticles in Au-NP-S-BPP network on a TEM grid substrate.

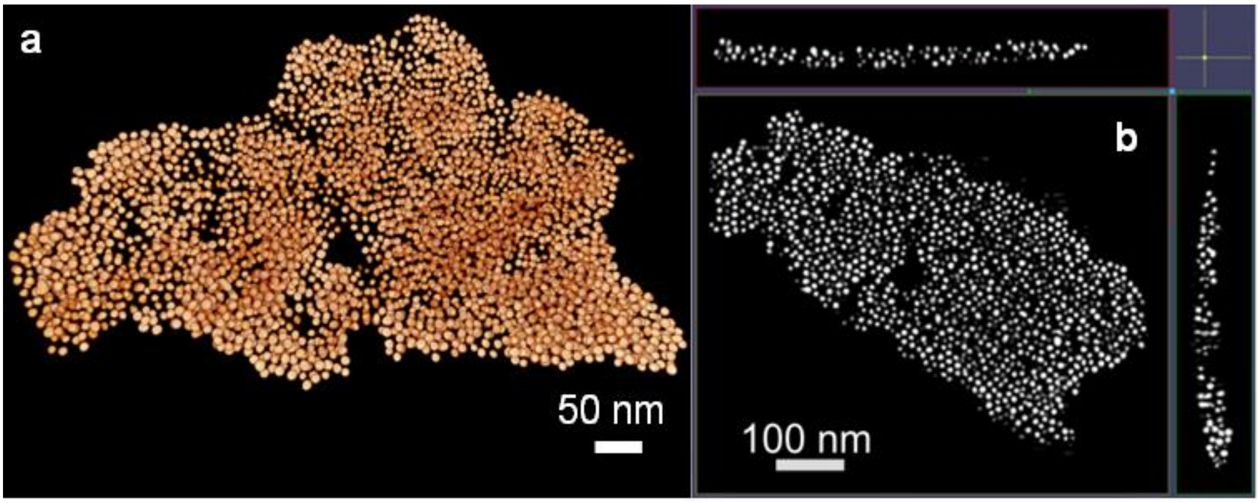

Figure S7: a) Volume rendering of the 3D electron tomographic reconstruction of a multilayered Au-NP-S-BPP network (on a TEM grid substrate) showing the packed ordering via sterically coordinating BPP molecules of a 3D metal-nanoparticle network. b) Digital slices along three orthogonal directions show the clear separation of the metal nanoparticles in the Au-NP-S-BPP network also in the 3rd dimension.

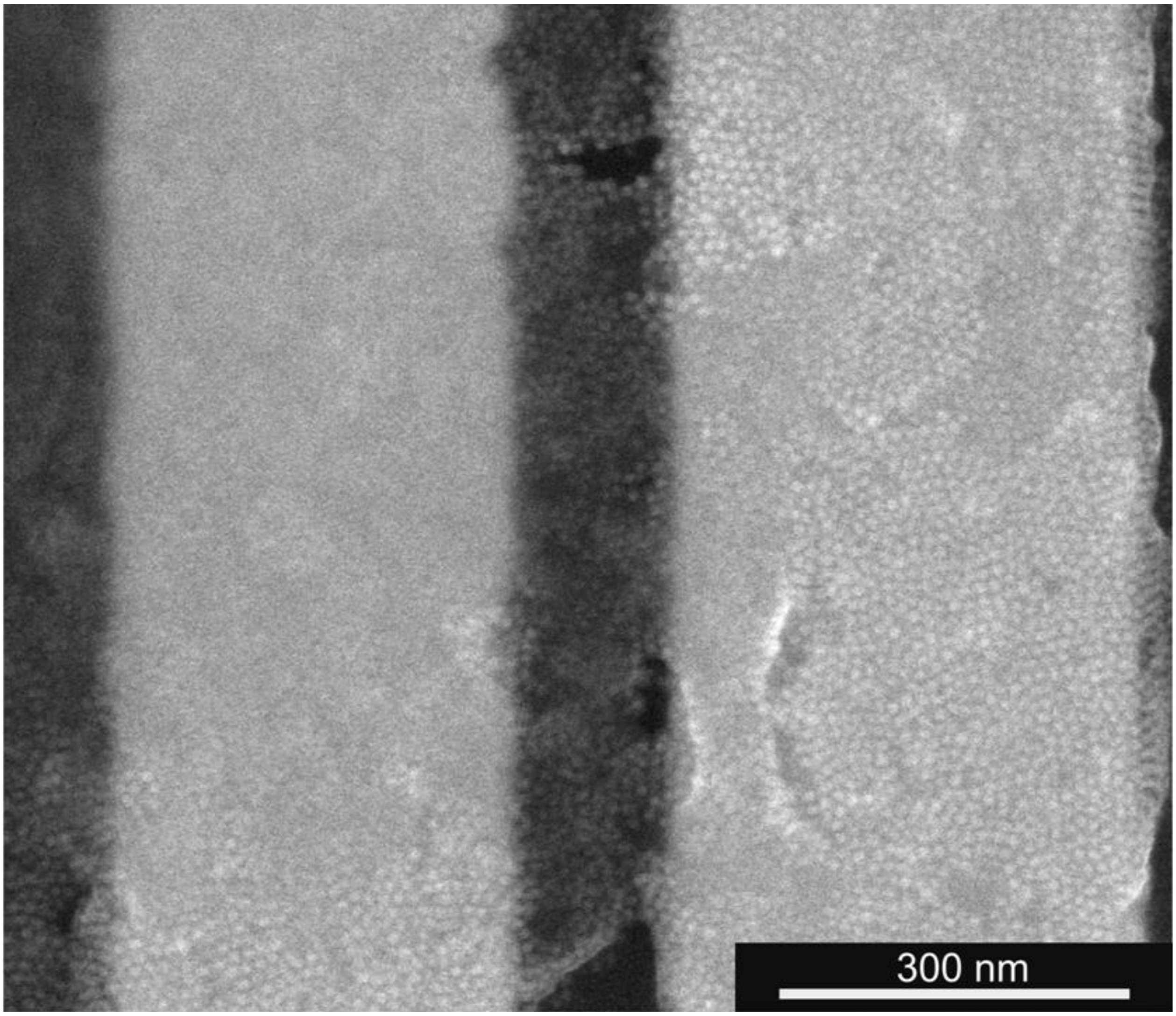

Figure S8: SEM image of Au-NP-S-BPP network device for conductance experiments. The two Au electrodes have been defined by electron-beam lithography. The resulting 'nanotrenches' of around 120 nm distance between the Au electrodes ensure a favourable aspect ratio for conductance measurements. These Au-NP-S-BPP networks have been obtained via three microcontact printing sequences of Au-NP-S-BPP arrays onto the nanotrench devices. In general, the networks are reasonably well ordered. However, single defects and larger voids (as seen in the image) will be present too. Although the latter may lead to more complicated percolation paths, conductance properties are expected to be dominated by the more ordered regions. We also note that ordering on the SiO_2 and on the Au nanotrench

electrodes is similar. The main reason for this is that the Au-NP-S-BPP arrays were self-assembled on water, before they were actually transferred to a substrate.

3. Spectroscopic characterisation of alkanethiol-gold nanoparticles and S-BPP-gold nanoparticle dispersions

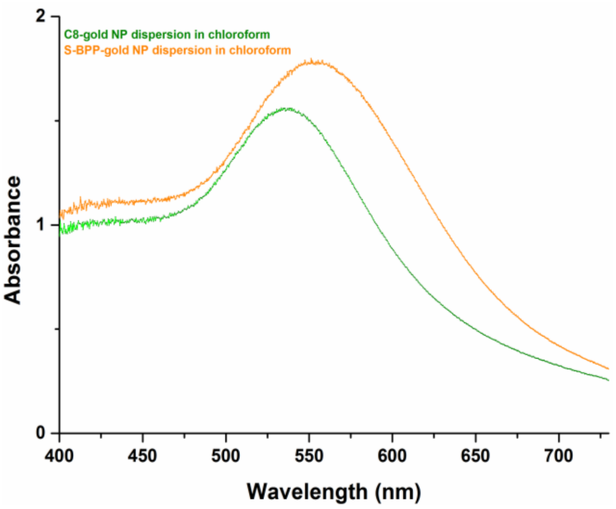

Figure S9: Surface plasmon resonance spectroscopy of (-) C8 functionalized gold nanoparticle dispersion versus (-) S-BPP-gold nanoparticle dispersion. For both dispersions a chloroform solvent is used.

4. Surface enhanced Raman spectroscopic characterisation of alkanethiol-gold nanoparticles array

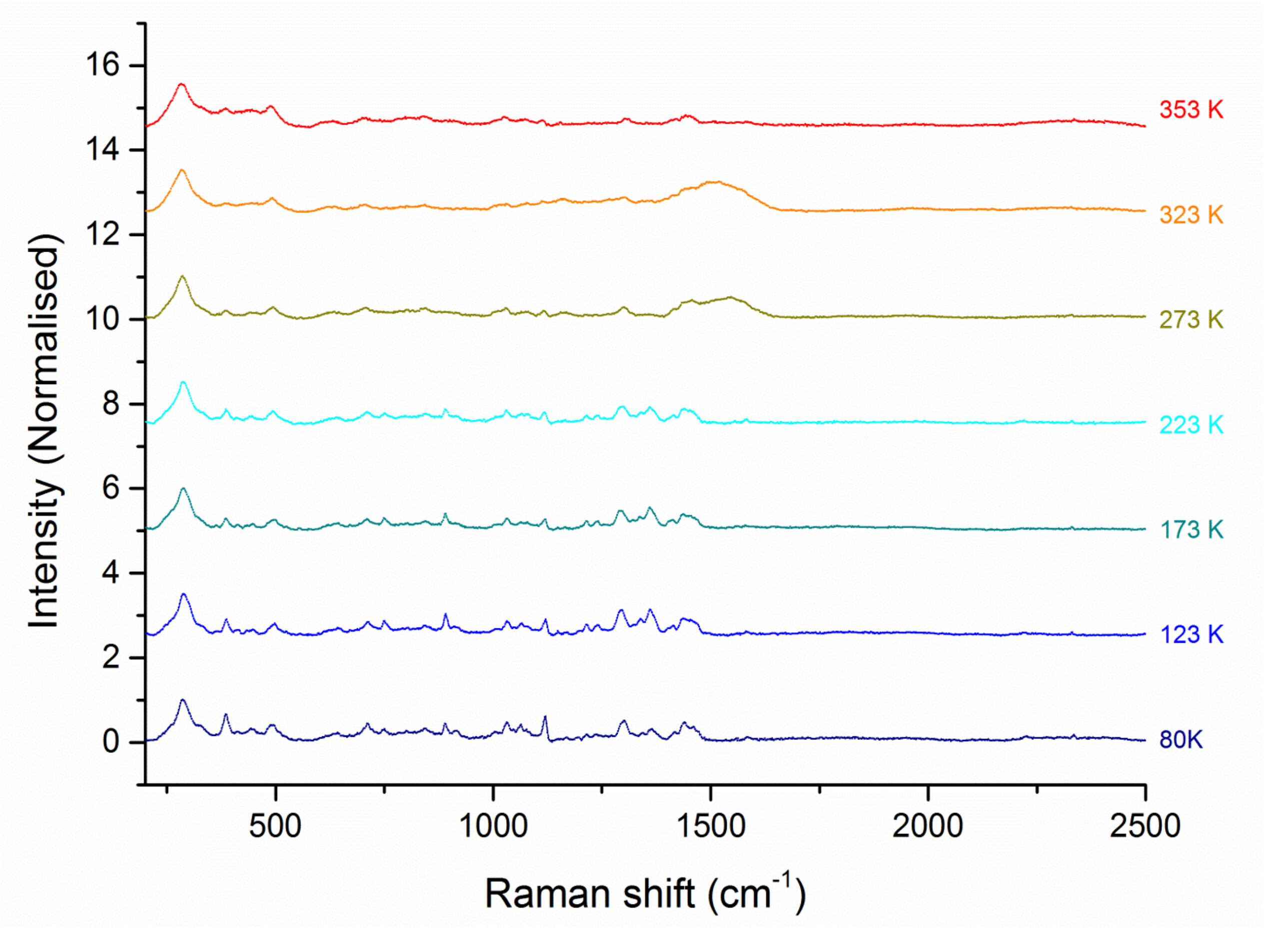

Figure S10: Temperature dependent Raman spectra of C8-gold nanoparticle array, microcontact printed on a quartz substrate excited at 633 nm (illuminating power 1.2 mW at the sample).

5. Charge transport data: temperature dependent I-V's of a multilayered Au-NP-S-BPP network

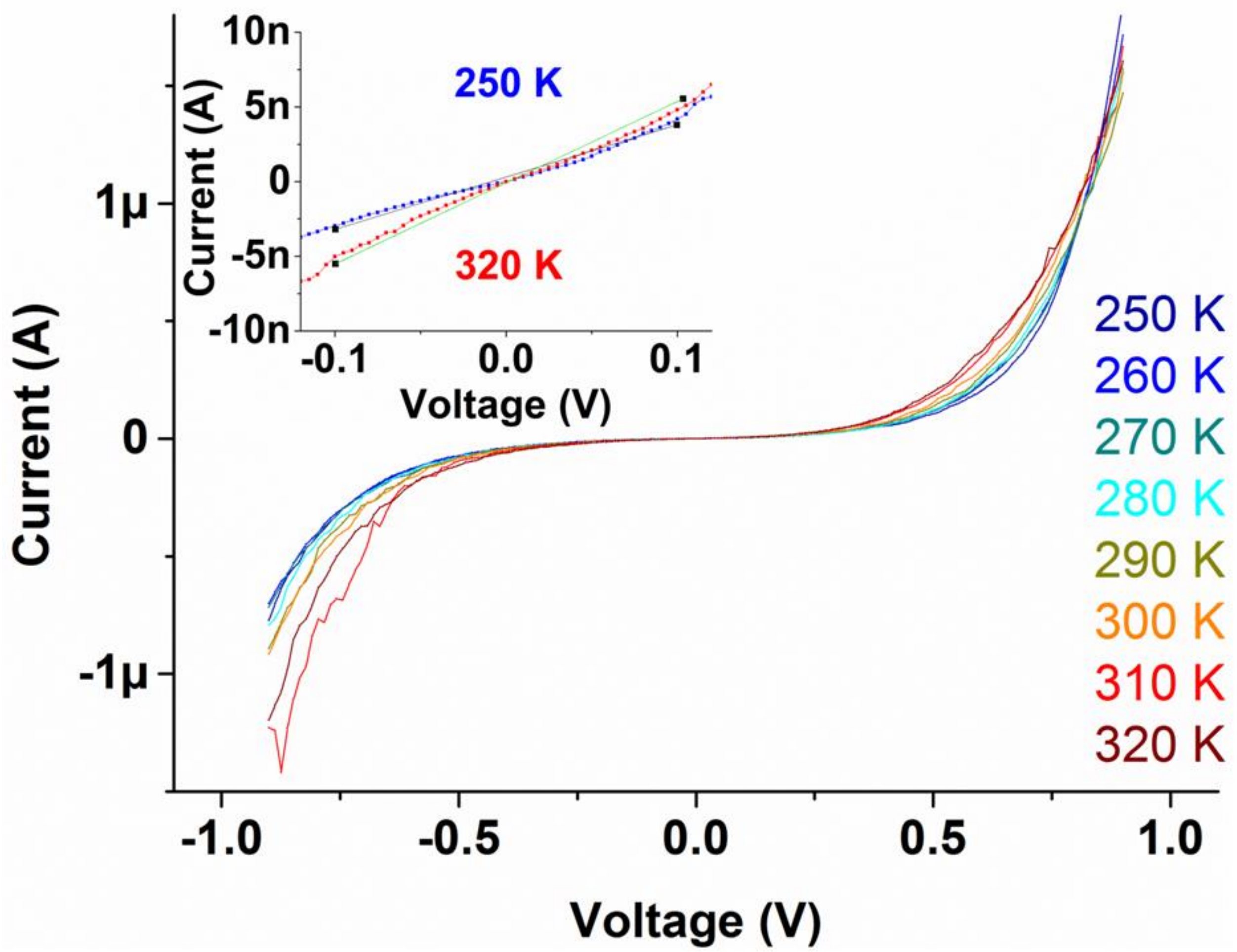

Figure S11: Temperature dependent I-V curves of a multilayered Au-NP-S-BPP network on a HAR nanotrench device. The data are taken between 250 K and 320 K (the inset zooms in at low bias range for the I-V curves at 250 K and 320 K).

Charge transport data: temperature dependant resistance of a C8-gold nanoparticle network and an OPE-gold nanoparticle network

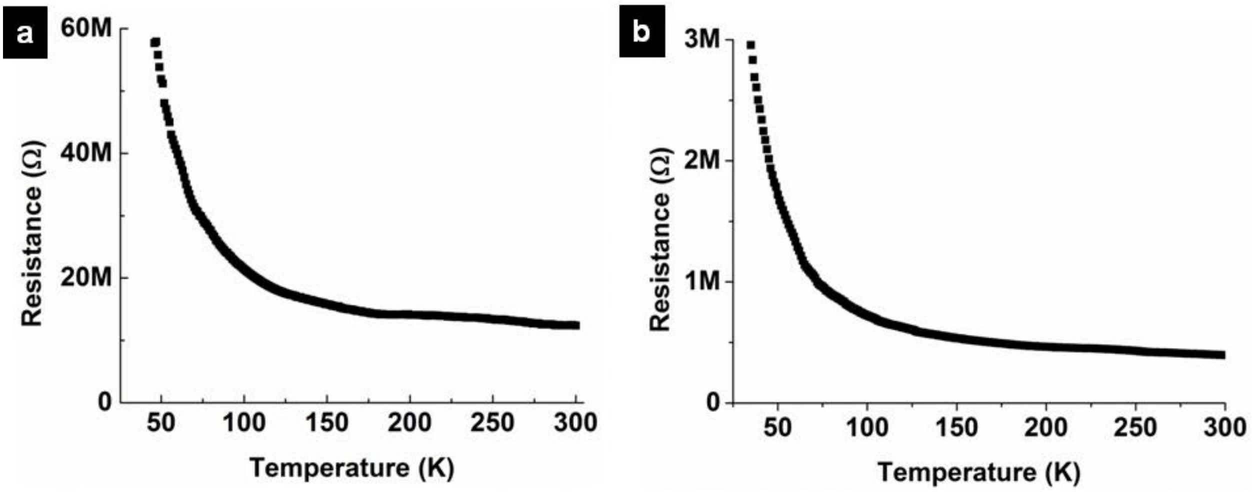

Figure S12: a) Low-bias resistance as function of temperature plot of a multilayered (three times stamped) C8-gold nanoparticle network on HAR nanotrench device. b) Low-bias resistance as function of temperature plot of a multilayered (three times stamped) OPE network (i.e. a C8-gold nanoparticle network exchanged with bridging OPE molecules) on HAR nanotrench device.

6. Modelling the charging energy of 2D Au-NP-S-BPP arrays as function of the dielectric constant

To get a feeling for the expected values of E_C for these multilayered Au-NP-S-BPP networks, we plot E_C in Figure S13 as a function of the dielectric constant based on three models to approximate the E_C for a 2D Au-NP-S-BPP array.

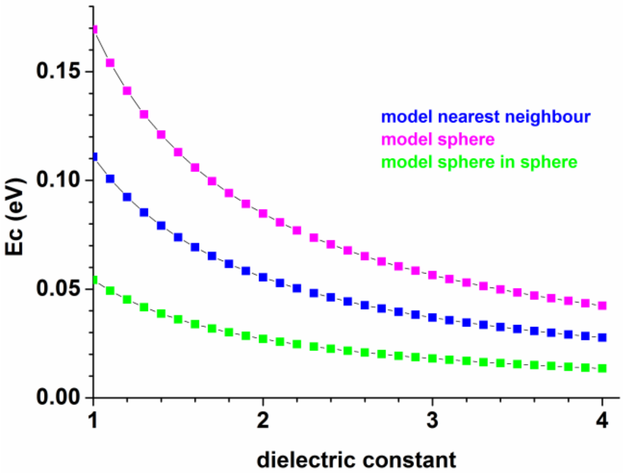

Figure S13: E_C as function of the dielectric constant for a 2D Au-NP-S-BPP array, calculated through three different models.

The following models are used in Figure S13 to determine the E_C of a 2D Au-NP-S-BPP array. The first model is the simple spherical model to calculate the maximum limit of E_C for a 2D Au-NP-S-BPP array (see equation S1).

$$Ec = \frac{e^2}{8\pi\varepsilon_0\varepsilon_r r} \qquad \text{(S1)}$$

Where r is the radius "r" of the gold nanoparticles in a 2D Au-NP-S-BPP array (earlier determined by TEM of value 0.5*8.5 nm). The dielectric constant ε of a 2D Au-NP-S-BPP array (based on UV-Vis analyses) is set on 2.8.

The second model is called the “sphere in sphere” model which estimates a minimum limit of E_C. In the model “sphere in sphere” the total capacitance of a gold nanoparticle is calculated assuming it is fully surrounded by other gold nanoparticles. This is approximated by assuming that the metallic sphere is in a second concentric metal shell [6] (see equation S2).

$$Ec = \frac{e^2}{\frac{8\pi\varepsilon_0\varepsilon_r}{\left(\frac{1}{r}-\frac{1}{r+d}\right)}} \quad \text{(S2)}$$

Where for “d” (the length of two entangled S-BPP molecules) is used 2 nm. The radius “r” remains set 4.25 nm.

The third model is called the “nearest neighbour” model [7] (see equation S3).

$$Ec = \frac{e^2}{2\left([4\pi\varepsilon_0\varepsilon_r r]+\left[4\pi\varepsilon_0\varepsilon_r\frac{r^2}{d}\left(1+\frac{r^2}{d^2-2r^2}+\frac{r^4}{d^4-4d^2r^2+3r^4}\right)\right]\right)} \quad \text{(S3)}$$

Based on mirror charges between 2 spherical nanoparticle, this model calculates an E_C intermediate value that fits between the upper and lower E_C limits of respectively equation 5 and 6. Here r is the radius of the gold NP in 2D Au-NP-S-BPP array and d is 2r+2nm (the distance between two nanoparticles separated by BPP molecules with a dielectric constant of 2.8).

From Figure S13 we determine that for 2D Au-NP-S-BPP array with a dielectric constant of 2.8, the E_C of 2D Au-NP-S-BPP array ranges 0.019-0.060 eV. Unfortunately, the models are rather crude so, a good quantitative comparison to the data is not possible. Note however that the experimental value of E_C is expected to

be significantly larger for alkanethiol arrays than for Au-NP-S-BPP arrays, due to the lower dielectric constant in the alkanethiol arrays (see UV-Vis section in article).

References

1. Slot J.; Geuze, H. *Eur. J. Cell Biol.* **1985**, *38*, 87-93. (PMID:4029177)
2. Chandrasekar, R.; Schramm, F.; Fuhr, O.; Ruben, M. *Eur. J. Inorg. Chem.* **2008**, *17*, 2649-2653. doi:10.1002/ejic.200800212
3. Huang, S.; Tsutsui, G.; Sakaue, H.; Shingubara, S.; Takahagi, T. *J. Vac. Sci. Technol. B* **2001**, *19*, 2045-2049. doi:10.1116/1.1410943
4. Santhanam, V.; Andres, R. P. *Nano Lett.* **2004**, *4*, 41-44. doi:10.1021/nl034851r
5. Dayen, J.-F.; Faramarzi, V.; Pauly, M.; Kemp, N. T.; Barbero, M.; Pichon, B. P.; Majjad, H.; Begin-Colin, S.; Doudin B. *Nanotechnology* **2010**, *21,* 335303. doi:10.1088/0957-4484/21/33/335303
6. Noguchi, Y.; Terui, T.; Katayama, T.; Matsushita, M. M.; and Sugawara, T. *Appl. Phys. Lett.* **2011**, *98*, 263114-263117. doi:10.1063/1.3605598
7. Allen, M. *Matter, Geometry, and Electromagnetics*, **2006**, S-*96.4620*, 1-9.
